# SPOP point mutations regulate substrate preference and affect its function

**DOI:** 10.1038/s41419-024-06565-1

**Published:** 2024-02-26

**Authors:** Yanran Deng, Wenhao Ding, Kaize Ma, Meixiao Zhan, Li Sun, Zizhang Zhou, Ligong Lu

**Affiliations:** 1 Jiangsu Key laboratory of Drug Screening, China Pharmaceutical University, 210009 Nanjing, China; 2 Key Laboratory of Biodiversity Conservation and Bioresource Utilization of Jiangxi Province, College of Life Sciences, Jiangxi Normal University, 330022 Nanchang, China; 3 College of Life Sciences, Shandong Agricultural University, 271018 Tai’an, China; 4 Guangdong Provincial Key Laboratory of Tumor Interventional Diagnosis and Treatment, Zhuhai People’s Hospital (Zhuhai Hospital Affiliated with Jinan University), 519000 Zhuhai, Guangdong China

**Keywords:** Tumour-suppressor proteins, Metastasis

## Abstract

The adaptor SPOP recruits substrates to CUL3 E3 ligase for ubiquitination and degradation. Structurally, SPOP harbors a MATH domain for substrate recognition, and a BTB domain responsible for binding CUL3. Reported point mutations always occur in SPOP’s MATH domain and are through to disrupt affinities of SPOP to substrates, thereby leading to tumorigenesis. In this study, we identify the tumor suppressor IRF2BP2 as a novel substrate of SPOP. SPOP enables to attenuate IRF2BP2-inhibited cell proliferation and metastasis in HCC cells. However, overexpression of wild-type SPOP alone suppresses HCC cell proliferation and metastasis. In addition, a HCC-derived mutant, SPOP-M35L, shows an increased affinity to IRF2BP2 in comparison with wild-type SPOP. SPOP-M35L promotes HCC cell proliferation and metastasis, suggesting that M35L mutation possibly reprograms SPOP from a tumor suppressor to an oncoprotein. Taken together, this study uncovers mutations in SPOP’s MATH lead to distinct functional consequences in context-dependent manners, rather than simply disrupting its interactions with substrates, raising a noteworthy concern that we should be prudent to select SPOP as therapeutic target for cancers.

## Introduction

The speckle-type pox virus and zinc finger (POZ) protein (SPOP) is a substrate-binding adaptor of the CUL3-RING E3 ubiquitin ligase complex [1]. SPOP protein contains two functional domains: the MATH domain in its N terminus and the BTB domain in its C terminus [1–3]. In general, the MATH domain recognizes diverse substrates, while the BTB domain scaffolds with the CUL3-RING complex, together leading to ubiquitination and subsequent degradation of substrates [1]. Thus, the MATH domain of SPOP confers its specificities to substrates. These years, increasing SPOP substrates have been identified, including GLI2, PD-L1, NANOG, TRIM24, CYCLIN E1 and c-MYC [4–9]. Since most of the reported substrates are oncoproteins, SPOP is considered as a tumor suppressor. However, in kidney cancers, SPOP promotes ubiquitin-mediated proteolysis of the anti-tumor PTEN and leads to tumorigenesis [10]. Small molecule disrupting the interaction between SPOP and PTEN is able to inhibit cancer cell proliferation [11]. Therefore, SPOP plays oncogenic or anti-tumor roles depending on its substrates in distinct cancers. Exploring SPOP-interacting substrates is necessary to gain insight into their role in tumorigenesis and to evaluate their feasibility as therapeutic target.

Another evidence of a tight link between SPOP and tumorigenesis comes from its high frequency of mutation in human tumor samples. Exon sequencing identifies SPOP mutations in up to 13% of prostate cancer (PC) samples, ranking first among all mutated genes [12]. Knockdown of SPOP in PC cells enhances invasive ability, pointing to its tumor-suppressive role [12]. Mechanistically, mutations cause SPOP to lose its inherent ability to ubiquitinate the oncoproteins, such as ERG and SRC3, leading to tumorigenesis in PCs [13, 14]. Another study has found high frequencies of somatic mutations in SPOP in endometrial cancers (ECs) [15]. Remarkably, all somatic mutations are clustered in the MATH domain of SPOP, suggesting that mutations possibly influence the interactions between SPOP and substrates. Since SPOP mutations in PCs and ECs occur on different sites, these mutations may bring about distinct outcomes. In line with this, PC-derived SPOP mutants fail to bind and ubiquitinate the oncoprotein DEK, while EC-derived mutants retain an equivalent ability to ubiquitinate DEK as wild-type SPOP [16]. On the contrary, EC-associated mutations in SPOP weaken its affinity to IRF1, but PC-derived mutations do not [17]. The crystal structure shows that the MATH domain of SPOP forms a hydrophobic cleft for substrate recognition and interaction [3]. Mutations in the cleft may affect its selection for substrates. Most reported SPOP mutations in the MATH domain have been defined as loss-of-function, due to the reduced or lost affinities to substrates. Whether the MATH mutations bring about gain-of-function effects, even result in novel substrates, is still unclear. The gain-of-function mutations will lead SPOP to acquire new functions or reprogram its activity.

Interferon regulatory factor 2-binding protein 2 (IRF2BP2) has been originally identified as a transcription corepressor of interferon regulatory factor-2 (IRF-2) that regulates the expression of various genes related to oncogenic processes, including cell proliferation, metastasis, and immune response [18–22]. Recent studies have uncovered that IRF2BP2 cooperates with transcriptional suppressor VGLL4 to inhibit YAP-TEAD4 activity, thereby suppressing the progression of YAP-induced liver cancer [21]. In addition, VGLL4 enhances the stability of IRF2BP2, and loss of VGLL4 results in persistent binding of IRF2 to PD-L1 promoter and reduces its expression [18]. In zebrafish liver, deficiency of irf2bp2a, the ortholog of human IRF2BP2, induces apoptosis through promoting p53 destabilization [23]. Furthermore, IRF2BP2 also regulates a variety of cellular functions in breast cancer, leukemia, and chondrosarcoma [24–26]. IRF2BP2 interacts with the proapoptotic NRIF3 to control Caspase-2-dependent cell death in breast cancer cells [27]. In acute myeloid leukemia cells, IRF2BP2 modulates inflammation and cell death by suppressing the canonical NF-κB pathway [26]. In addition, recent studies have demonstrated that the gene fusion of IRF2BP2 and others is possibly an inducer for tumorigenesis. The IRF2BP2-RARA gene fusion leads to resistance to all-trans retinoic acid chemotherapy in patients with acute promyelocytic leukemia [28, 29]. The IRF2BP2-CDX1 gene fusion is involved in the progression of mesenchymal chondrosarcomas [24]. Although growing studies have revealed the important anti-tumor role of IRF2BP2 in various cancers, the mechanisms for governing its abundance are still unclear.

To identify SPOP-interacting partners, we carried out immunoprecipitation using SPOP as bait and subsequent mass spectrometry assays and found IRF2BP2 as a candidate. In this study, we employed biochemical experiments to validate that IFR2BP2 is a bona fide substrate of SPOP. SPOP bound IRF2BP2 via its N-terminal MATH domain. In IRF2BP2, we identified a matched SPOP binding consensus (SBC), whose mutation would disrupt its interaction with SPOP. SPOP promoted IRF2BP2 ubiquitination in a CUL3-dependent manner. Functionally, IRF2BP2 suppressed the proliferation and migration of HCC cells, which was rescued by co-expression of SPOP. However, overexpression of IRF2BP2 alone also inhibited HCC cell proliferation and migration, reflecting its anti-tumor role. Furthermore, PC-derived mutations abolished SPOP binding to IRF2BP2, whereas EC-derived mutations failed to affect its affinity with IRF2BP2. Finally, we identified an HCC-derived mutant, SPOP-M35L, that showed increased interaction with IRF2BP2. Consistently, SPOP-M35L exhibited more robust activity to ubiquitinate and degrade IRF2BP2. In contrast to wild-type SPOP, SPOP-M35L enabled to promote of HCC cell proliferation and migration possibly due to its high affinity to IRF2BP2. Thus, the M35L mutation reprogrammed SPOP from a tumor suppressor to an oncoprotein. To our knowledge, this is the first time a point mutation was discovered that enables to reverse of the tumor-related properties of SPOP.

## Results

### SPOP binds IRF2BP2 through its N-terminal MATH domain

**Fig. 1:**
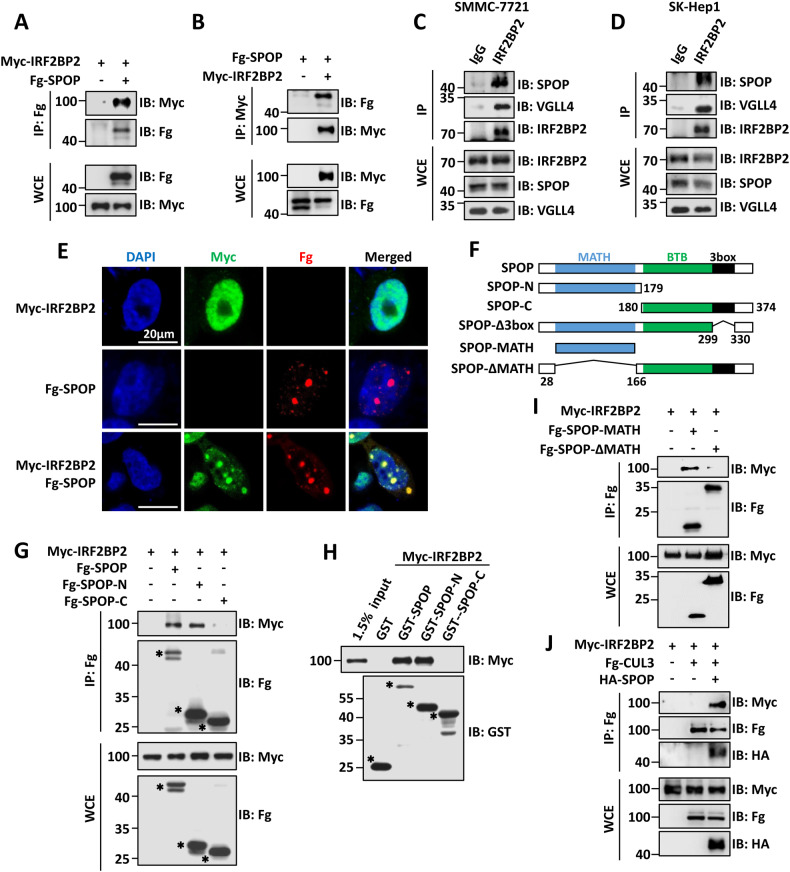
SPOP binds IRF2BP2 through its N-terminal MATH domain. **A** Immunoblots of immunoprecipitates (IP, top two panels) or whole cell extracts (WCE, bottom two panels) from HEK-293T cells transfected with indicated plasmids and treated with 25 μM of MG132 for 8 h. Of note, Fg-SPOP could pull down Myc-IRF2BP2. **B** Myc-IRF2BP2 was able to immunoprecipitate Fg-SPOP in HEK-293T cells. **C** and **D** Endogenous IRF2BP2 interacted with endogenous SPOP in SMMC-7721 cells (**C**) and SK-Hep1 cells (**D**). **E** HEK-293T cells transfected with indicated constructs were stained to show Myc-IRF2BP2 (green), Fg-SPOP (red) and DAPI (blue). DAPI staining marks the cell nuclei. Scale bars: 20 μm for all images. **F** Schematic drawings showed the domains in SPOP and the truncated mutants used in the following studies. **G** Both Fg-SPOP and Fg-SPOP-N could, but Fg-SPOP-C could not pull down Myc-IRF2BP2. Asterisks mark the SPOP truncated fragments. **H** Extracts from HEK-293T cells overexpressing Myc-IRF2BP2 were incubated with GST or indicated GST fusion proteins. The bound proteins were analyzed by western blot. Asterisks mark GST fusion proteins. **I** Fg-SPOP-MATH interacted with Myc-IRF2BP2 in HEK-293T cells. **J** Fg-CUL3 enabled to pull down Myc-IRF2BP2 in the presence of HA-SPOP.

To explore novel SPOP-interacting proteins, we performed immunoprecipitation using SPOP as bait and subsequent mass spectrometry experiments and found IRF2BP2 as a candidate. The co-immunoprecipitation (co-IP) result showed that Fg-SPOP indeed pulled down Myc-IRF2BP2 in HEK-293T cells (Fig. 1A). Reciprocally, Myc-IRF2BP2 also immunoprecipitated Fg-SPOP (Fig. 1B). Given that IRF2BP2 exhibits potent tumor-suppressor activity in the hepatocellular carcinoma (HCC) [21], we explored whether IRF2BP2 is an authentic SPOP substrate primarily in liver cancer cells. The co-IP result showed that the endogenous IRF2BP2 could pull down endogenous SPOP and VGLL4, a previously reported IRF2BP2 interactor [18], in SMMC-7721 and SK-Hep1 cells (Fig. 1C, D). In addition, we carried out immunofluorescence (IF) analyses to examine the co-localization of Myc-IRF2BP2 and Fg-SPOP proteins. As shown in Fig. 1E, when Fg-SPOP or Myc-IRF2BP2 was transfected alone, Fg-SPOP showed a speckle-like distribution and Myc-IRF2BP2 evenly localized in the nucleus, while Myc-IRF2BP2 co-localized into Fg-SPOP speckles in the nucleus when co-transfected with Fg-SPOP.

SPOP contains a MATH domain in its N-terminus, which is always responsible for binding substrates, and a BTB domain in its C-terminal region which is important for its CUL3-binding and self-oligomerization [1, 2]. To determine which domain in SPOP is responsible for its interaction with IRF2BP2, we divided SPOP into two parts named SPOP-N and SPOP-C (Fig. 1F). The co-IP results revealed that only the SPOP-N enabled to pull down IRF2BP2 (Fig. 1G). The glutathione-S-transferase (GST) pull-down assay further validated this result (Fig. 1H). To examine the importance of the MATH domain for SPOP binding IRF2BP2, we generated Fg-SPOP-MATH and Fg-SPOP-ΔMATH truncated constructs (Fig. 1F). The co-IP results showed that Fg-SPOP-MATH could, but Fg-SPOP-ΔMATH could not pull down Myc-IRF2BP2 (Fig. 1I), suggesting that the MATH domain is sufficient and necessary for SPOP binding to IRF2BP2. As SPOP primarily functions as a substrate-recognizing subunit of the SPOP-CUL3 E3 ligase complex [1], we then tested whether CUL3, SPOP and IRF2BP2 form a ternary complex. The co-IP data showed that Fg-CUL3 could immunoprecipitate Myc-IRF2BP2 in presence of HA-SPOP (Fig. 1J). Taken together, SPOP interacts with IRF2BP2 using its MATH domain and recruits CUL3 to IRF2BP2.

### IRF2BP2 interacts with SPOP via a classical SBC motif

**Fig. 2:**
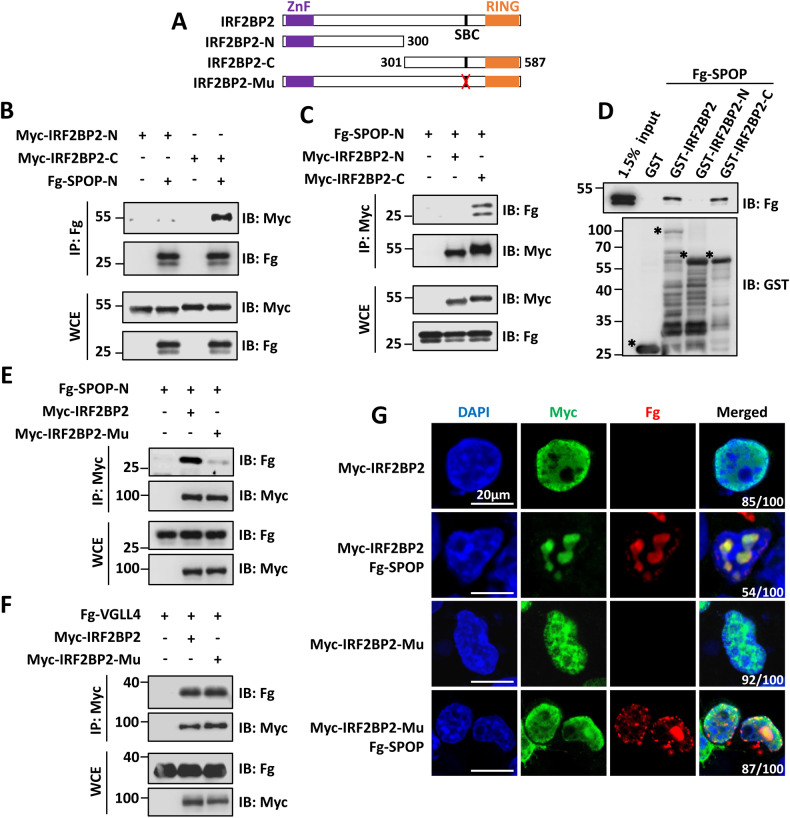
SPOP interacts with IRF2BP2 by recognizing its SBC motif. **A** Schematic representation of the domains and SBC motif in IRF2BP2 protein and their truncated mutants used in the following experiments. **B** and **C** Immunoblots of immunoprecipitates (top two panels) or whole cell extracts (bottom two panels) from HEK-293T cells transfected with indicated plasmids and treated with 25 μM of MG132 for 8 h. Of note, Fg-SPOP-N interacts with Myc-IRF2BP2-C, not Myc-IRF2BP2-N. **D** Extracts from HEK-293T cells overexpressing Fg-SPOP were incubated with GST or indicated GST fusion proteins. The bound proteins were analyzed by western blot. Asterisks mark GST fusion proteins. **E** Mutation of SBC motif in IRF2BP2 abolished it binding to SPOP-N in HEK-293T cells. **F** SBC motif-mutated form of IRF2BP2 could bind VGLL4 in HEK-293T cells. **G** HEK-293T cells expressing indicated constructs were stained to show Myc-IRF2BP2/IRF2BP2-Mu (green), Fg-SPOP (red) and DAPI (blue). DAPI staining marks the cell nuclei. The percentage of cells in each group exhibiting the indicated phenotype was shown at the lower right corner of merged plot. Scale bars: 20 μm for all images.

IRF2BP2 protein harbors two conserved domains: a zinc finger domain in its N terminus and a RING domain in its C terminus [22]. To map the SPOP-interacting region, two IRF2BP2 truncated fragments (Fig. 2A) were co-transfected with SPOP-N into HEK-293T cells respectively, followed by co-IP assays. Results showed that IRF2BP2-C reciprocally pulled down SPOP-N, whereas IRF2BP2-N did not (Fig. 2B, C). We next confirmed these results through GST pull-down assay (Fig. 2D). The previous study has demonstrated that the MATH domain of SPOP always recognizes a N-P-S-S/T-S/T (N-nonpolar, P-polar) motif, named SPOP-binding consensus (SBC) [1]. Increasing studies have shown that most SPOP substrates contain at least one SBC, such as GLI2, NANOG and c-MYC [4, 6, 9]. Therefore, we set out to search IRF2BP2 protein sequence and discovered one perfectly matched SBC (^447^VHST^451^T) just localized in IRF2BP2-C (Fig. 2A). To explore the importance of this SBC for SPOP–IRF2BP2 interaction, we substituted VHSTT with alanines to generate Myc-IRF2BP2-Mu construct (Fig. 2A). Compared with Myc-IRF2BP2, Myc-IRF2BP2-Mu exhibited apparently weak binding to Fg-SPOP-N (Fig. 2E). However, Myc-IRF2BP2-Mu and Myc-IRF2BP2 showed equivalent affinity to Fg-VGLL4 (Fig. 2F), inferring that the SBC mutation specifically abolishes SPOP–IRF2BP2 association. IF results further confirmed that Fg-SPOP exclusively co-localized with Myc-IRF2BP2, not Myc-IRF2BP2-Mu in nuclear speckles (Fig. 2G). Collectively, IRF2BP2 harbors a matched SBC motif in its C terminus, which is essential for its interaction with SPOP.

### SPOP promotes proteasome-mediated IRF2BP2 proteolysis

**Fig. 3:**
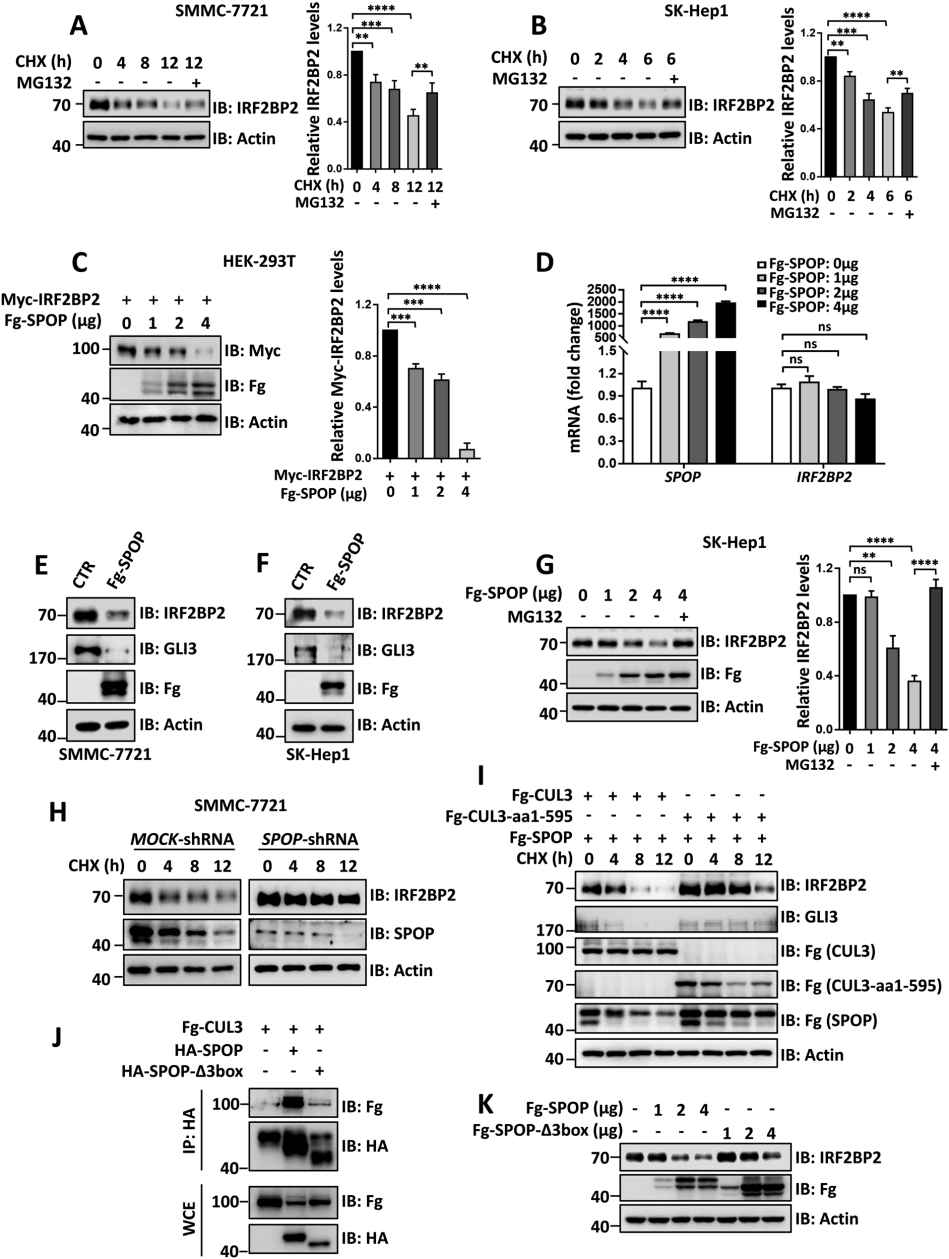
SPOP promotes IRF2BP2 proteasomal degradation. **A** Protein levels of IRF2BP2 in SMMC-7721 cells after treated with CHX for indicated hours with or without MG132 treatment for 4 h prior cell harvesting. The relative levels of IRF2BP2 normalized to Actin were shown on the right. **B** Protein levels of IRF2BP2 from SK-Hep1 cells with indicated treatment. Quantitative analyses were shown on the right. **C** SPOP decreased Myc-IRF2BP2 protein in a dose-dependent manner in HEK-293T cells. **D** Real-time PCR to examine the mRNA levels. **E** and **F** Overexpression of SPOP decreased endogenous IRF2BP2 and GLI3 protein levels in SMMC-7721 cells (**E**) and SK-Hep1 cells (**F**). **G** SPOP degraded endogenous IRF2BP2 in SK-Hep1 cells. Quantitative analyses were shown on the right. (**H**) Knockdown of SPOP retarded endogenous IRF2BP2 degradation. **I** CUL3-aa1-595 inhibited SPOP-mediated IRF2BP2 degradation. GLI3 acts as a positive control. **J** 3box deletion in SPOP attenuated it binding to CUL3 in HEK-293T cells. **K** Protein levels of IRF2BP2 from HEK-293T cells transfected with different amount of Fg-SPOP or Fg-SPOP-Δ3box. For all results, Actin acts as a loading control. For statistical analyses, data are means ± SEM. from three biological-independent experiments. In all above, **P < 0.01, ***P < 0.001, ****P < 0.0001, ns: not significant difference based on student’s *t*-test.

Since our above data clearly demonstrated that IRF2BP2 is an interacting partner of SPOP, we next sought to test whether SPOP promotes IRF2BP2 degradation. At first, we treated HCC cells with translation inhibitor cycloheximide (CHX) to block de novo protein synthesis for distinct durations and detected IRF2BP2 protein via immunoblotting. The results showed that IRF2BP2 protein was unstable, with a half-life of about 4 h in SMMC-7721 cells (Fig. 3A) and SK-Hep1 cells (Fig. 3B). The proteasome inhibitor MG132 could partially upregulate IRF2BP2 protein levels (Fig. 3A, B), indicating that IRF2BP2 is degraded, at least in part, through the proteasome. Subsequently, we found that Fg-SPOP enabled to decrease Myc-IRF2BP2 in a dose-dependent manner, without influencing *IRF2BP2* mRNA levels (Fig. 3C, D).

To further investigate the effect of SPOP on endogenous IRF2BP2, we ectopically expressed SPOP in HCC cells and found that endogenous IRF2BP2 and GLI3 (a well-characterized SPOP substrate) protein levels were reduced obviously (Fig. 3E, F). Moreover, SPOP promoted endogenous IRF2BP2 degradation in a dose-dependent manner, which could be blocked by MG132 treatment (Fig. 3G). In contrast, the knockdown of SPOP by shRNA in SMMC-7721 apparently extended the half-life of IRF2BP2 protein in comparison with *MOCK*-shRNA (Fig. 3H). Given that SPOP often ubiquitinates substrates with the help of CUL3, we examined whether CUL3 is required for SPOP-mediated degradation of IRF2BP2. As a matter of fact, CUL3 acts as a platform to recruit E2 and adaptors, followed by transferring ubiquitin from E2 to adaptor-linked substrates [30]. We generated a truncated construct, Fg-CUL3-aa1-595, whose E2-binding region was deleted. Previous studies have demonstrated that CUL3-aa1-595 plays a dominant-negative role, possibly due to competing with endogenous CUL3 to bind adaptors [31, 32]. In line with this, co-expressed with SPOP and CUL3-aa1-595 apparently slowed down the degradation of IRF2BP2 and GLI3 (Fig. 3I). On the other hand, we employed SPOP-Δ3box mutant, which the CUL3 binding region (3box) in SPOP was deleted to abolish its interaction with CUL3 [1]. Compared with SPOP, SPOP-Δ3box exhibited apparently weak binding to CUL3 and lower ability to degrade endogenous IRF2BP2 (Fig. 3J, K), together suggesting that SPOP degrades IRF2BP2 through SPOP-CUL3 complex. In summary, as SPOP could promote exogenous and endogenous IRF2BP2 degradation in a variety of different HCC cell lines, SPOP-mediated proteolysis is a universal mechanism for IRF2BP2 degradation. The degradation ability of SPOP to IRF2BP2 and the half-life of IRF2BP2 in different cell lines may be decided by SPOP abundance.

### SPOP promotes K48-linked polyubiquitination of IRF2BP2

**Fig. 4:**
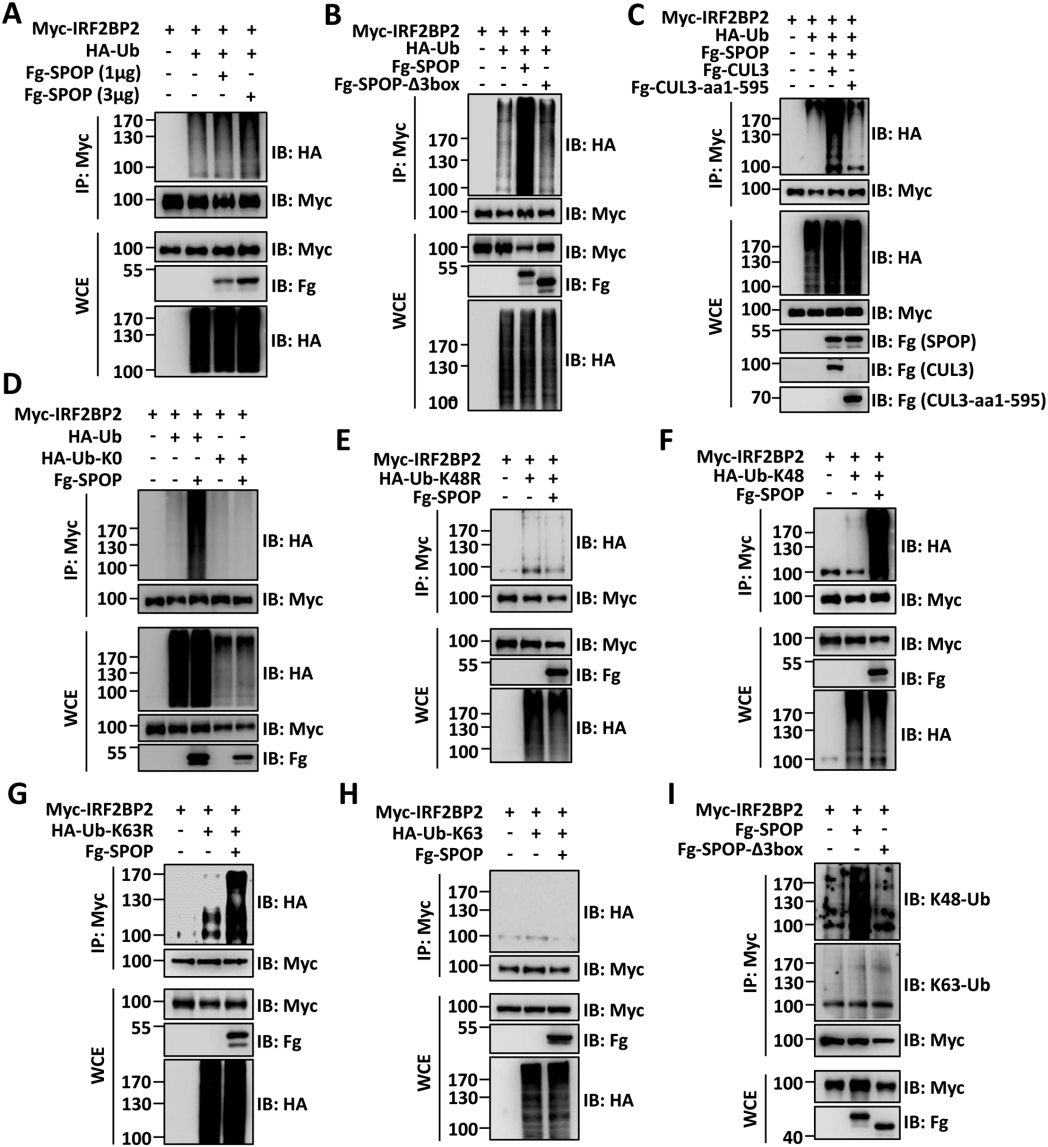
SPOP catalyzes K48-linked polyubiquitination of IRF2BP2 in a CUL3-dependent manner. **A** Immunoblots of immunoprecipitates (top two panels) or whole cell extracts (bottom three panels) from HEK-293T cells expressing indicated plasmids and treated with MG132 for 6 h before cell harvesting. Overexpression of SPOP promoted IRF2BP2 ubiquitination in a dose-dependent manner. **B** SPOP-Δ3box failed to induce IRF2BP2 ubiquitination. **C** CUL3 promoted IRF2BP2 ubiquitination, while CUL3-aa1-595 could not. **D** SPOP enhanced polyubiquitination of IRF2BP2. **E** and **F** SPOP promoted K48-linked polyubiquitination on IRF2BP2. **G** and **H** SPOP did not promote K63-linked polyubiquitination on IRF2BP2. **I** SPOP could, but SPOP-Δ3box could not, promote K48-linked polyubiquitination on IRF2BP2.

Given that SPOP binds IRF2BP2 and promotes IRF2BP2 degradation, it needs to examine whether SPOP directly ubiquitinates IRF2BP2. The ubiquitination assays revealed that SPOP promoted the ubiquitination of IRF2BP2 in a dose-dependent manner (Fig. 4A). Compared with SPOP, SPOP-Δ3box failed to trigger IRF2BP2 ubiquitination (Fig. 4B), suggesting that CUL3 is essential for SPOP-induced ubiquitination of IRF2BP2. Consistently, SPOP could not ubiquitinate IRF2BP2 in the present of CUL3-aa1-595 (Fig. 4C). Ubiquitin-mediated protein degradation can occur through monoubiquitination and polyubiquitination [33, 34]. To examine the type of IRF2BP2 ubiquitination, we generated Ub-K0, in which all lysines were mutated to arginines, preventing polyubiquitin chain formation [35]. As shown in Fig. 4D, SPOP failed to ubiquitinate IRF2BP2 in the presence of Ub-K0, pointing to polyubiquitination occurring on IRF2BP2.

For polyubiquitination, the first ubiquitin (Ub) covalently links to the lysine (K) of substrates [33]. The following Ub adds to one of the lysines of the prior Ub to gradually form a polyubiquitin chain [33]. Although Ub has seven lysines (K6, K11, K27, K29, K33, K48 and K63), K48 and K63 are the main recipients for Ub [36]. To examine which linkage polyubiquitination occurs on IRF2BP2, we employed Ub-K48R and Ub-K63R, in which the respective lysine was replaced by arginine. The co-IP results showed that SPOP-induced ubiquitination of IRF2BP2 was eliminated in the presence of Ub-K48R (Fig. 4E), while Ub-K63R did not affect the SPOP-mediated IRF2BP2 ubiquitination (Fig. 4G). Contrarily, the Ub-K48, which harbors only one K on 48, has a similar effect as wild-type Ub on SPOP-mediated IRF2BP2 ubiquitination (Fig. 4F). However, SPOP failed to ubiquitinate IRF2BP2 in the presence of Ub-K63 (Fig. 4H). Furthermore, we used K48-Ub and K63-Ub antibodies to specifically detect K48- and K63-linked polyubiquitin chains respectively, and found SPOP, not SPOP-Δ3box promoted K48-linked ubiquitination on IRF2BP2 (Fig. 4I).

Although SPOP binds to SBC in IRF2BP2-C (Fig. 2B–D), SPOP failed to degrade IRF2BP2-C (Fig. S1A), indicating that the ubiquitination sites possibly localize in the N terminus of IRF2BP2. To figure out which lysines are responsible for IRF2BP2 ubiquitination, we carried out a prediction using online software [37], and identified eight potential sites in IRF2BP2-N (K60, K82, K89, K164, K236, K257, K259 and K289). We mutated each of these sites and tested the degradative effect of SPOP on these mutants, and found four mutants (K236R, K257R, K259R and K289R) are resistant to SPOP-mediated degradation (Fig. S1B). The following ubiquitination assays demonstrated that K236R or K289R mutations apparently suppressed SPOP-induced IRF2BP2 ubiquitination (Fig. S1C). In addition, SPOP failed to degrade IRF2BP2-K236R (Fig. S1D), even stabilized IRF2BP2-K289R possibly due to its domain-negative role (Fig. S1E). Accordingly, K236R and K289R mutations did not abolish IRF2BP2 binding to SPOP (Fig. S1F), thus the degradative defects could not be attributed to loss of binding. Furthermore, we employed Myc-IRF2BP2-K236/289R, which harbors both of the above mutations. The chase experiments revealed that Myc-IRF2BP2-K236/289R escaped to be degraded in the presence of Fg-SPOP (Fig. S1G). Meanwhile, dose-gradient expression of Fg-SPOP failed to induce Myc-IRF2BP2-K236/289R degradation (Fig. S1H), together suggesting that K236/289 are key ubiquitination sites. Taken together, with the assistance of CUL3, SPOP promotes K48-linked polyubiquitination on K236 and K289 of IRF2BP2, leading to proteasome-mediated degradation.

### SPOP counteracts the anti-proliferation role of IRF2BP2

**Fig. 5:**
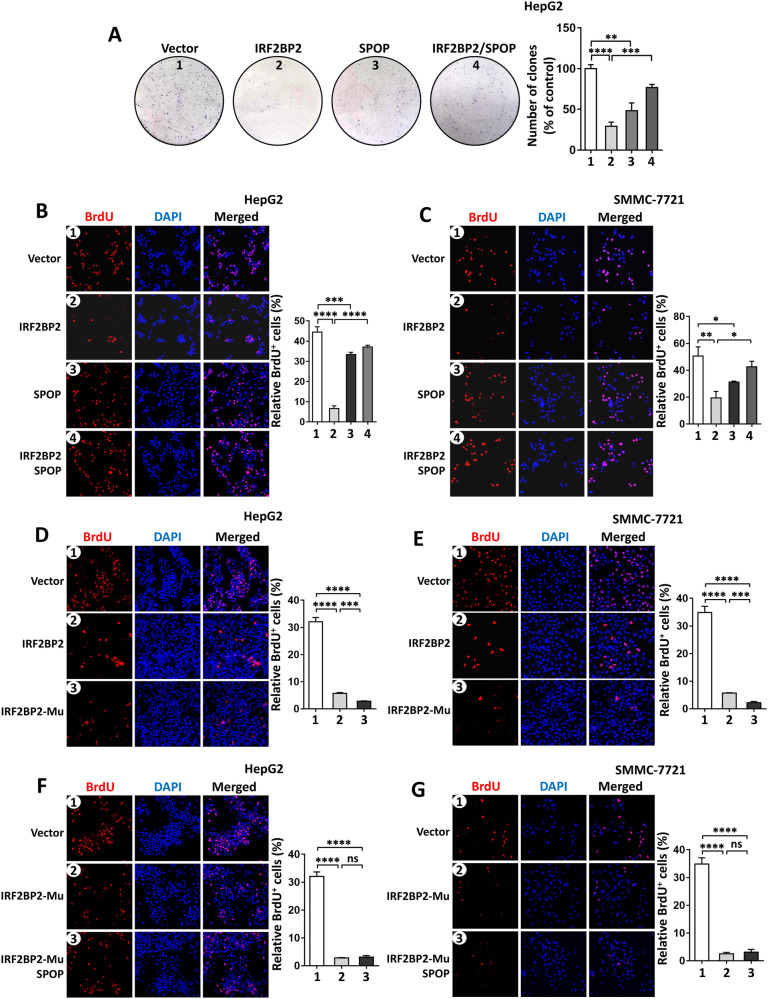
SPOP rescues IRF2BP2-mediated inhibition on HCC cell proliferation. **A** The colony formation assay showed the proliferative ability of HepG2 cells with indicated transfection. Quantitative analyses were shown on the right. **B** and **C** The BrdU incorporation assays revealed that IRF2BP2-induced cell cycle arrest were restored by SPOP co-expression in HepG2 cells (**B**) and SMMC-7721 cells (**C**). Quantification analyses were shown on the right. **D** and **E** Compared with IRF2BP2, IRF2BP2-Mu showed stronger ability to suppress HepG2 (**D**) and SMMC-7721 (**E**) cell proliferation. Quantitative analyses were shown on the right. **F** and **G** SPOP failed to restore the proliferation suppression induced by IRF2BP2-Mu. Quantitative analyses were shown on the right. For statistical analyses, data are means ± SEM from three biological-independent repeats. In all above, *P < 0.05, **P < 0.01, ***P < 0.001, ****P < 0.0001, ns: not significant difference based on student’s *t*-test.

Previous studies have demonstrated that IRF2BP2 binds VGLL4 to boost VGLL4’s inhibitory effect on YAP, therefore suppressing HCC progression [21]. Due to our observations of SPOP-promoting ubiquitination-mediated IRF2BP2 degradation, we next explored the biological significance of the SPOP–IRF2BP2 axis in HCC tumorigenesis. Consistently, forced expression of IRF2BP2 did inhibit the colony formation of SK-Hep1 cells, reflecting the attenuated proliferation (Fig. S2A). In contrast, the silence of IRF2BP2 using shRNA promoted colony formation in SMMC-7721 cells (Fig. S2B). In addition, we used the BrdU incorporation assay to evaluate the activity of cell division. As expected, IRF2BP2 overexpression diminished, while IRF2BP2 knockdown enhanced BrdU signals in HCC cells (Fig. S2C, D). In HepG2 cells, IRF2BP2 suppressed colony formation, which was rescued by SPOP co-expression (Fig. 5A). Surprisedly, overexpression of SPOP alone also inhibited colony formation (Fig. 5A). On the other hand, IRF2BP2 enabled to decrease BrdU incorporation in HepG2 and SMMC-7721 cells, which was effectively restored by SPOP co-transfection (Fig. 5B, C). In consistent with colony formation, SPOP alone was capable of suppressing BrdU incorporation (Fig. 5B, C). Accordingly, the knockdown of SPOP obviously elevated BrdU incorporation in HepG2 (Fig. S3C) and SMMC-7721 cells (Fig. S3D), together demonstrating its anti-proliferative role. These results raise a seemingly contradictory conclusion that the tumor-suppressive SPOP attenuates IRF2BP2-mediated tumor inhibition. To further validate whether the inhibition of IRF2BP2 by SPOP affected the activity of YAP-TEAD, we carried out RT-PCR assays to test YAP target genes and found that overexpression of IRF2BP2 indeed down-regulated the expression levels of YAP target genes, which were rescued by SPOP co-expression (Fig. S3G). In addition, we confirmed this result through luciferase analyses, in which a DNA fragment containing an Hpo-responsive element (*HRE*-Luc) was used to respond to YAP-TEAD activity. IRF2BP2 decreased *HRE*-Luc activity, which was rescued by SPOP co-expression (Fig. S3H).

Given that SPOP binds the SBC in IRF2BP2, we next examined whether disruption of the interaction boots IRF2BP2’s anti-tumor activity. As expected, IRF2BP2-Mu showed more stability than wild-type IRF2BP2 (Fig. S3A), and could not be ubiquitinated by SPOP (Fig. S3B). Compared with wild-type IRF2BP2, IRF2BP2-Mu exhibited a stronger inhibitory effect on BrdU incorporation in HepG2 (Fig. 5D) and SMMC-7721 cells (Fig. 5E), possibly due to its resistance to SPOP-mediated degradation. Supportively, the inhibition of BrdU incorporation induced by IRF2BP2-Mu failed to be restored by SPOP co-transfection (Fig. 5F, G). Overall, albeit SPOP plays an anti-proliferative role in HCC cells, it is able to relieve IRF2BP2-induced cell proliferation inhibition by promoting IRF2BP2 degradation.

### SPOP antagonizes the anti-migration ability of IRF2BP2

**Fig. 6:**
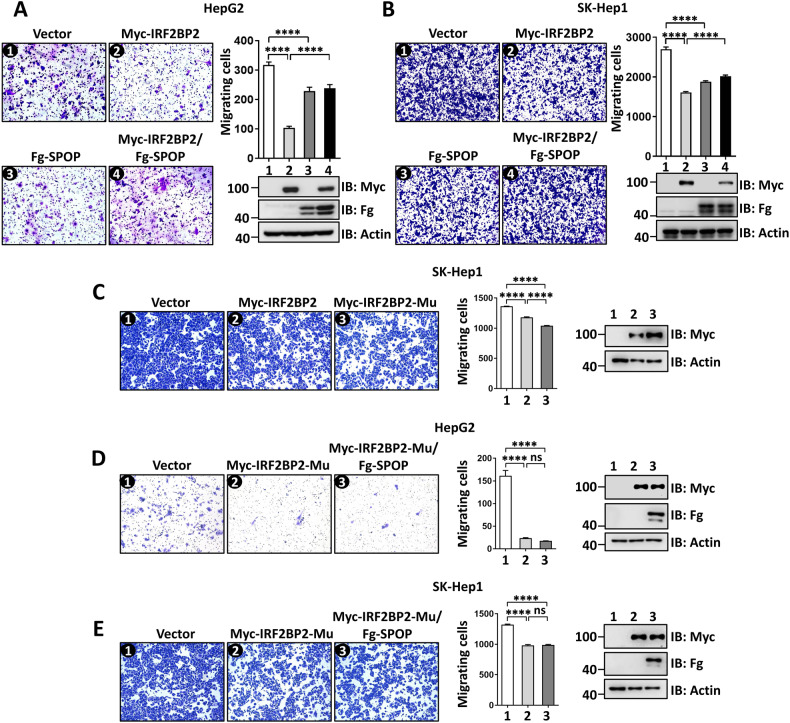
SPOP rescues IRF2BP2-induced inhibition of HCC cell migration. **A** The transwell assays showed the migration of HepG2 cells transfected with indicated constructs. The expressions of constructs were examined using immunoblotting. Quantitative analyses were shown on the right. **B** SPOP restored IRF2BP2-mediated inhibition of SK-Hep1 cell migration. The expressions of constructs were detected by immunoblotting. Quantitative analyses were shown on the right. **C** Compared with IRF2BP2, IRF2BP2-Mu showed stronger ability to suppress SK-Hep1 cell migration. Protein expressions and quantitative analyses were shown on the right. **D** and **E** Co-transfection of SPOP cannot rescue IRF2BP2-Mu-mediated migration suppression of HepG2 cells (**D**) and SK-Hep1 cells (**E**). Protein expressions and quantitative analyses were shown on the right. For all immunoblotting data, Actin acts as a loading control. For statistical results, data are shown as means ± SEM from three biological-independent repeats. In all above, ***P < 0.001, ****P < 0.0001, ns: not significant difference based on student’s *t*-test.

Besides, transwell assays demonstrated that overexpression of IRF2BP2 inhibited SK-Hep1 cell migration (Fig. S2E), while IRF2BP2 knockdown promoted SMMC-7721 cell migration (Fig. S2F), suggesting that IRF2BP2 is a negative regulator for cell migration. IRF2BP2-induced cell migration suppression in HepG2 and SK-Hep1 cells was rescued by SPOP co-transfection (Fig. 6A, B). In line with cell proliferation, overexpression of SPOP alone suppressed cell migration (Fig. 6A, B). In contrast, the silence of SPOP promoted cell migration (Fig. S3E, F), reflecting its anti-migration role in HCC cells. In SK-Hep1 cells, IRF2BP2-Mu exerted stronger inhibition on cell migration than IRF2BP2 (Fig. 6C). Consistently, the suppression of cell migration by IRF2BP2-Mu failed to be remitted by SPOP co-transfection (Fig. 6D, E). Taken together, SPOP enables suppress IRF2BP2-induced suppression of HCC cell migration via targeting IRF2BP2 for degradation.

### Tumor-derived mutations of SPOP differentially reprogram its activity in a context-dependent manner

Systematic sequencing studies have demonstrated that SPOP presents high-frequency mutations in human cancer samples, especially in prostate cancer [12] and endometrial cancer [15]. Remarkably, most mutations are clustered in the MATH domain (Fig. S4A-B). Increasing studies uncovered that tumor-derived SPOP mutations disrupt SPOP interacting with substrates, preventing their degradation [5–9]. We next sought to test whether tumor-derived mutations affect SPOP interactions with IRF2BP2. The co-IP and ubiquitination results showed that PC-derived SPOP mutants (Y87N, S119N, F125I, K129E and F133V) did not pull down and ubiquitinate IRF2BP2 (Fig. S4C and S5E), whereas EC-derived SPOP mutants (E47K, M117I and R121Q) comparably immunoprecipitated and ubiquitinated IRF2BP2 as wild-type SPOP (Fig. S4D and S5F). Similar to wild-type SPOP, PC- and EC-derived SPOP mutants displayed speckle distribution in cell nuclei (Fig. S4E), suggesting that these mutations do not alter SPOP sub-cellular localization. In consistent with the co-IP results, EC-derived SPOP mutants could recruit Myc-IRF2BP2 to speckles, but PC-derived SPOP mutants could not (Fig. S4E). Functionally, PC-derived SPOP mutants disabled to rescue IRF2BP2-caused cell proliferation arrest (Fig. S5A, B), while EC-derived SPOP mutants showed equal suppression as wild-type SPOP on IRF2BP2’s cell anti-proliferation activity (Fig. S5C, D). These results suggest that SPOP mutations from distinct types of human cancer possibly produce differential outcomes.

**Fig. 7:**
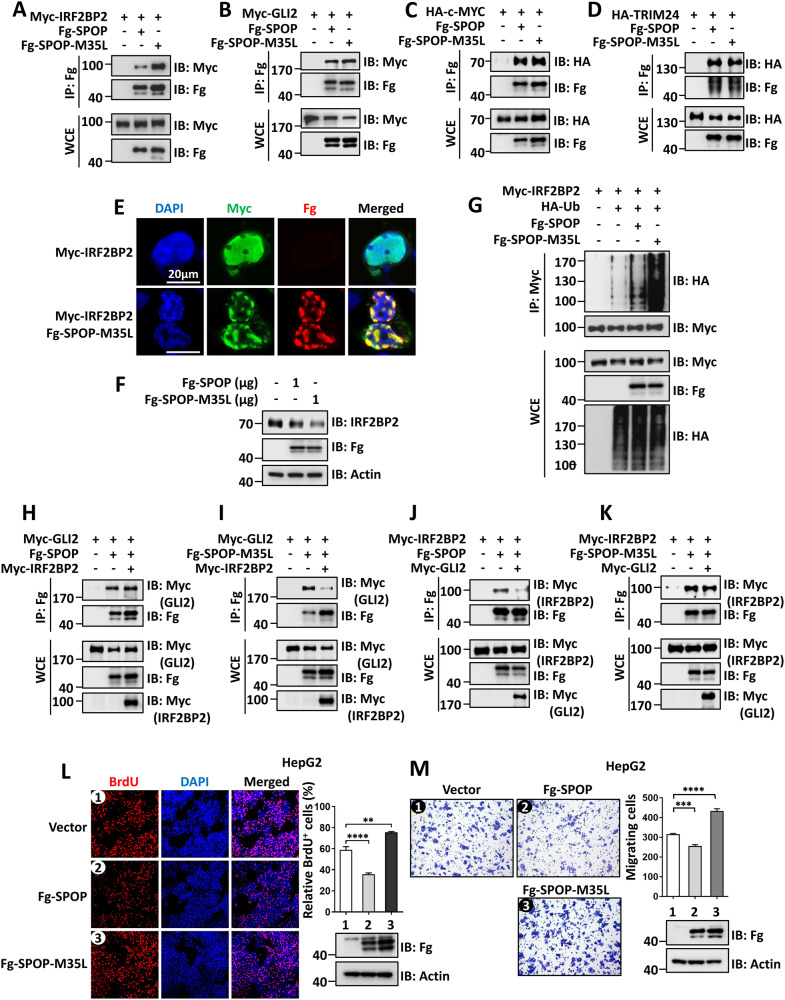
Identification a gain-of-function mutation in SPOP to enhance its interaction with IRF2BP2. **A** Compared with Fg-SPOP, Fg-SPOP-M35L showed stronger affinity to Myc-IRF2BP2. **B**–**D** Fg-SPOP and Fg-SPOP-M35L showed equal affinities to Myc-GLI2 (**B**), HA-c-MYC (**C**) and HA-TRIM24 (**D**). **E** HEK-293T cells expressing indicates constructs were stained to show Myc-IRF2BP2 (green), Fg-SPOP-M35L (red) and DAPI (blue). Scale bars: 20 μm for all images. **F** Protein levels of IRF2BP2 from HEK-293T cells transfected with equal amount of Fg-SPOP or Fg-SPOP-M35L. Actin acts as a loading control. **G** The ubiquitination levels of Myc-IRF2BP2 induced by Fg-SPOP-M35L or Fg-SPOP. **H** IRF2BP2 did not affect SPOP-GLI2 interaction. **I** IRF2BP2 weakened the interaction between SPOP-M35L and GLI2. **J** GLI2 decreased SPOP-IRF2BP2 interaction. **K** GLI2 failed to influence the binding of SPOP-M35L and IRF2BP2. **L** The BrdU incorporation assays of HepG2 cells expressing SPOP or SPOP-M35L. Quantification analysis was shown on the right. **M** The transwell assays HepG2 cells expressing SPOP or SPOP-M35L. Protein expressions and quantitative analyses were shown on the right. Actin acts as a loading control. For statistical results, data are shown as means ± SEM from three biological-independent experiments. In all above, **P < 0.01, ***P < 0.001, ****P < 0.0001, ns: not significant difference based on Student’s *t*-test.

Having demonstrated that SPOP mutations exert distinct effects dependently on cancer types, we set out to explore HCC-related SPOP mutations. Using the Cancer Genome Atlas (TCGA), we amazingly found two point mutations, M35L and F136L, in several liver cancer samples. Considering that M35 and F136 just localize in the MATH domain, we first examined whether these two mutations influence SPOP-IRF2BP2 interaction. Compared with wild-type SPOP, SPOP-M35L showed higher affinity to IRF2BP2 (Fig. 7A), suggesting M35L enhances SPOP binding to IRF2BP2. However, the M35L mutation did not affect SPOP interaction with other substrates including GLI2 (Fig. 7B), c-MYC (Fig. 7C) and TRIM24 (Fig. 7D), pointing to the specific effect of this mutation. In addition, we validated that SPOP-M35L enabled to anchor Myc-IRF2BP2 to nuclear speckles (Fig. 7E), confirming the interaction between IRF2BP2 and SPOP-M35L. In line with the observations in protein interaction, SPOP-M35L robustly degraded endogenous IRF2BP2 (Fig. 7F). Consistently, SPOP-M35L showed a stronger ability to ubiquitinate IRF2BP2 in comparison with wild-type SPOP (Fig. 7G). In contrast, compared with wild-type SPOP, SPOP-F136L showed equal affinity and degradation ability to IRF2BP2 (Fig. S6G, H).

Since the above results showed that M35L mutation specifically enhances SPOP affinity to IRF2BP2, it is necessary to investigate whether this somatic mutation affects SPOP substrate selectivity. As shown, IRF2BP2 did not alter the interaction between GLI2 and wild-type SPOP (Fig. 7H), while decreased GLI2 binding to SPOP-M35L (Fig. 7I). On the other hand, GLI2 weakened IRF2BP2 binding to wild-type SPOP (Fig. 7J), without attenuating the interaction between IRF2BP2 and SPOP-M35L (Fig. 7K). These results suggested that wild-type SPOP prefers the oncoprotein GLI2, while SPOP-M35L favors the tumor-suppressor IRF2BP2. In addition, we explored whether this regulation is applied to other substrates. The co-IP results showed that IRF2BP2 did not decrease SPOP binding to c-MYC (Fig. S6A) or TRIM24 (Fig. S6C), but attenuated SPOP-M35L interaction with c-MYC (Fig. S6B) or TRIM24 (Fig. S6D). Based on these, we propose the hypothesis that M35L mutation possibly exchanges SPOP substrates from oncoproteins (such as GLI2, c-MYC and TRIM24) to tumor suppressors (such as IRF2BP2), therefore reprogramming the tumor properties of SPOP. Indeed, wild-type SPOP inhibited, while SPOP-M35L promoted the proliferation of HepG2 cells (Fig. 7L). Furthermore, wild-type SPOP suppressed HepG2 cell migration, while SPOP-M35L enhanced cell migration (Fig. 7M), together suggesting that M35L mutation likely reprograms SPOP from a tumor antagonist to a tumor promotor via substrate selection.

Although several studies have uncovered the anti-tumor role of SPOP in HCC [38–40], the latest paper shows that SPOP protein levels have no changes in human HCC samples [41]. We collected 16 pairs of HCC samples (cancer, C) and corresponding paracancerous samples (normal, N) for immunoblotting. The results revealed that normal and cancer samples expressed comparable SPOP (Fig. S6E). Additionally, the chase experiments showed that SPOP-M35L exhibited comparable stability to wild-type SPOP (Fig. S6F), indicating that M35L mutation only changes SPOP activity, not affecting its abundance. These results suggest that we cannot simply determine the oncogenic or anti-tumor role of a gene based on its expression. Taken together, our studies show that the M35L mutation reprograms SPOP from a tumor suppressor to a tumor promotor without affecting its protein level.

## Discussion

**Fig. 8:**
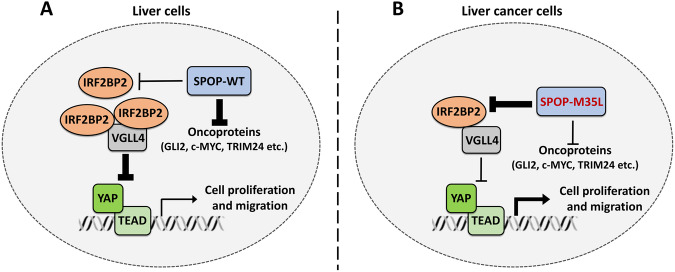
A proposed model for SPOP regulating IRF2BP2. **A** In normal liver cells, wild type SPOP prefers degrading oncoproteins, with weak affinity to IRF2BP2. In this circumstance, SPOP acts as a tumor suppressor. **B** In liver cancer cells, M35L mutation enhances SPOP interaction with IRF2BP2 to promote IRF2BP2 degradation. Thus, M35L mutation reprograms SPOP from a tumor suppressor to a oncoprotein.

Ubiquitination is an important mechanism for intercellular protein degradation, its dysregulation has been implicated in tumorigenesis. The E3 ligase determines which proteins are ubiquitinated by specifically binding to the substrates. Thus, the interaction between E3 ligases and substrates is decisive for ubiquitination. CUL-mediated E3 ligases are the largest E3 ligase family, in which CUL acts as a platform to recruit substrates using adaptors [42]. SPOP is a well-known adaptor for the CUL-based E3 ligase complex [3]. SPOP contains a MATH domain for recognizing substrates, and a BTB domain for binding CUL3 [1]. In past decades, increasing SPOP substrates have been identified, such as AR, GLI2, c-MYC, ERG, and TRIM24. Thus, SPOP is considered as a tumor suppressor. Systematical sequencing studies have revealed high-frequency mutations of SPOP in several types of human tumors, particularly in prostate and endometrial cancers [12, 15]. In addition, all identified somatic mutations are clustered in the MATH domain and display impaired substrate binding [12, 15]. In this study, we find the tumor suppressor IRF2BP2 as a novel substrate of SPOP. SPOP recognizes a classic SBC motif in IRF2BP2 using its MATH domain, leading to IRF2BP2 ubiquitination and subsequent degradation. Functionally, SPOP enables inhibition of the anti-tumor role of IRF2BP2 in HCC cells, which is eliminated by mutating the SBC motif in IRF2BP2. Furthermore, we identify a liver cancer-associated SPOP mutation, M35L, in its MATH domain. Surprisingly, M35L mutation enhances SPOP binding to IRF2BP2, rather than other substrates including GLI2, c-MYC and TRIM24. Finally, the M35L mutation reprograms SPOP from a tumor inhibitor to an oncoprotein, without affecting SPOP abundance. Compared with normal liver cells (Fig. 8A), SPOP-M35L shows a stronger affinity to IRF2BP2 in cancer cells (Fig. 8B), leading to the upregulation of cell proliferation and migration. To our knowledge, this study is the first time to provide a sample of a point mutation that is able to reverse the tumor-related functions of SPOP.

Structurally, SPOP comprises a MATH domain for substrate recognition and a BTB domain for binding CUL3. These two domains are dispensable for SPOP’s E3 ligase activity. However, exon sequencing reveals that tumor samples show high-frequency SPOP somatic mutations, which cluster in its MATH domain. Therefore, we cannot simply attribute these somatic mutations to disrupting SPOP E3 ligase activity, because mutations in the BTB domain can produce the same effect. In this study, we systematically analyze the effects of SPOP mutants derived from prostate cancers and endometrial cancers on IRF2BP2 and find the differential outcomes. Furthermore, we identify a novel point mutation M35L in HCC samples, which plays a gain-of-function role in tumorigenesis. Subsequent biochemical results show that the wild-type SPOP prefers binding oncoproteins (GLI2, c-MYC, etc.), while SPOP-M35L tends to interact with IRF2BP2. Thus, M35L mutation alters the substrate preference of SPOP, rather than disrupting its E3 ligase activity. In fact, HCC tissues and adjacent normal tissues express the same amount of SPOP, forming a so-called “SPOP pool”. The biological function of SPOP can be fine-tuned by adjusting its preference for substrates. Due to the close relationship between SPOP’s roles and its somatic mutations, the selection of SPOP as a therapeutic target requires full consideration of its mutations.

Although this study has clearly revealed that M35L mutation enhances SPOP binding to IRF2BP2, the underlying mechanism is unclear. A recent study analyzes the cryo-electron microscopic structure of SPOP and finds that E47K endometrial cancer mutation increases SPOP stability, while W22R endometrial cancer mutation alters its quaternary structure [43]. It will be fruitful to understand the gain-of-function role of SPOP-M35L by dissecting its structure. Besides binding substrates, the MATH domain is involved in SPOP high-order assembly, which is important for its E3 ligase activity. Whether the M35L mutation interferes with the high-order assembly of SPOP will be an interesting research direction. Further narrowing the amino acid residues in the MATH domain responsible for binding IRF2BP2 will facilitate to elucidate the molecular mechanism of IRF2BP2 recognition by SPOP.

## Materials and methods

### Cell lines and plasmids transfection

HEK-293T, HepG2, SMMC-7721 and SK-Hep1 cell lines were purchased from the ATCC. HEK-293T cells were maintained in Dulbecco’s modified Eagle’s medium (DMEM), and SMMC-7721 cells were maintained in RPMI-1640 medium supplemented with 10% fetal bovine serum (FBS) at 37 °C and 5% CO_2_. All these cell lines have been routinely tested to exclude the mycoplasma contamination and were transfected via lipo2000 (Thermo Fisher Scientific) according to the manufacturer’s instructions. For lentivirus transfection, pLKO.1-*MOCK* shRNA, pLKO.1-*SPOP* shRNA and pLKO.1-*IRF2BP2* shRNA plasmids were co-transfected with the packaging plasmids psPAX2 and pMD2G into HEK-293T cells using lipo3000 (Thermo Fisher Scientific). The infectious viral supernatant was collected 48 h after transfection. SMMC-7721 and HepG2 cells were infected with the viral supernatant, in the presence of 10 µg/mL polybrene (Hanbio Biotechnology). After being selected with 2 µg/mL puromycin for 2 weeks, all infected cells were tested using western blot.

### DNA constructs

The constructs for transfection were generated as follows: The IRF2BP2, SPOP, GLI2, VGLL4, CUL3, and Ub were amplified via PCR using HCC cells cDNA as the template and then cloned into the pcDNA3.1-Fg, pcDNA3.1-Myc or pcDNA3.1-HA backbone vectors. The truncated constructs including SPOP-N, SPOP-C, SPOP-MATH, SPOP-ΔMATH, SPOP-Δ3box, IRF2BP2-N, IRF2BP2-C, CUL3-aa1-595 were generated by inserting the indicated coding sequences into the corresponding backbone vectors. The point mutation constructs used in this study were created using PCR-based site-directed mutagenesis at the background of Fg-SPOP, Myc-IRF2BP2 or HA-Ub according to our previous study [37]. The *IRF2BP2*, *SPOP* and *MOCK* short hairpin RNAs were inserted into lentiviral vector PLKO.1-TRC. The target sequences used were as follows: *IRF2BP2*-shRNA, 5’-GCC GAC AGC CTG TCC ACC GCG-3’; *SPOP*-shRNA, 5’-GGT GCT ACA CAC AGA TCA AGG-3’; *MOCK*-shRNA, 5’-CAA CAA GAT GAA GAG CAC CAA-3’. For the *HRE*-Luc reporter, the TEAD recognition sequence, named Hippo responsive element (*HRE*) was amplified and inserted into the pGL3-Basic-Luc vector.

### Western blotting and immunofluorescence

48 h after transfection, cells were harvested and lysed for immunoblotting (IB) or immunoprecipitation (IP) analysis according to standard protocols [44]. The antibodies used were as follows: rabbit anti-SPOP (1:2000 for IB, 1:200 for IP; ProteinTech), rabbit anti-IRF2BP2 (1:1000 for IB, 1:100 for IP; ProteinTech), rabbit anti-GLI3 (1:1000 for IB; ABclonal), rabbit anti-VGLL4 (1:1000 for IB; ABclonal), rabbit anti-K48-Ub (1:1000 for IB; Abcam), rabbit anti-K63-Ub (1:1000 for IB; Abcam), mouse anti-Actin (1:5000 for IB; Genscript), mouse anti-Myc (1:2000 for IB, 1:200 for IP; Santa Cruz), mouse anti-Fg (1:5000 for IB, 1:500 for IP; Sigma) and mouse anti-HA (1:2000 for IB, 1:200 for IP; Santa Cruz).

For cell-based staining, transfected HEK-293T cells were plated on chamber slides and then fixed with 4% fresh-made formaldehyde for 20 min at room temperature. After washing three times with PBS, cells were permeabilized in 0.1% Triton X-100 in PBS for 10 min. Then cells were incubated with primary antibodies in PBS at 4 °C overnight. After washing with PBS, cells were incubated with fluorescence-labeled secondary antibody (1:500, Jackson ImmunoResearch) for 2 h and DAPI (1:1000, Sigma) for 20 min at room temperature in the dark. After rinsed in PBS three times, cells on slides were mounted with 40% glycerol, and images of cells were visualized and imaged using the Zeiss confocal microscope.

For BrdU incorporation assays, transfected SK-Hep1, HepG2 and SMMC-7721 cells were plated on chamber slides and incubated with 30 μM BrdU (Sigma) for 40 min before cell harvesting, and the subsequent immunofluorescence steps were carried out according to above described in these cells.

### GST fusion protein pull-down assay

GST pull-down assays were carried out as previously described [45]. Fusion proteins were induced expression by isopropyl β-D-thiogalactoside (IPTG) in *E. coli* BL21. Induced GST fusion proteins were purified using the Beaver beads GSH (Beaverbio) and then were incubated with cell lysates derived from HEK-293T cells containing corresponding proteins at 4 °C for 3 h. Finally, beads were washed three times and detected by WB assay.

### RNA extraction and qRT-PCR

Total RNAs were extracted from cultured cells with TRIzol (Psaitong) following standard protocols and reverse transcribed using HiScript® Q RT SuperMix with gDNA wiper (Vazyme) according to the manufacturer’s instructions. Quantitative real-time PCR was carried out on ZY/VQ-100A (Yuanzan) using the ChamQ SYBR® Color qPCR Master Mix (Vazyme). Relative expression of indicated genes was detected using the 2-∆∆Ct method. The primer pairs used were as follows: *SPOP*, 5’-GGT GCT ACA CAC AGA TCA AG-3’ (forward) and 5’-TAA TGA CTT CAC CCA TTT CC-3’ (reverse); *IRF2BP2*, 5’-CCC ATG ACT CCT ACA TCC TCT T-3’ (forward) and 5’-GAG GGC GGA CTG TTG CTA TTC-3’ (reverse); *CYR61*, 5’-CCT CGG CTG GTC AAA GTT AC-3’ (forward) and 5’-TTT CTC GTC AAC TCC ACC TC-3’ (reverse); *AREG*, 5’-TCA CTT TCC GTC TTG TTT TGG-3’ (forward) and 5’-CGG GAG CCG ACT ATG ACT AC-3’ (reverse); *CTGF*, 5’-AGG AGT GGG TGT GTG ACG A-3’ (forward) and 5’-CCA GGC AGT TGG CTC TAA TC-3’ (reverse); *ANKRD1*, 5’-AGT AGA GGA ACT GGT CAC TGG-3’ (forward) and 5’-TGG GCT AGA AGT GTC TTC AGA T-3’ (reverse); *ACTIN*, 5’-TGA CAT TAA GGA GAA GCT GTG CTA C-3’ (forward) and 5’-GAG TTG AAG GTA GTT TCG TGG ATG-3’ (reverse). Data are presented as means ± SEM of values from at least three experiments.

### Transwell assay

48 h after transfection, 1 × 10^5^ cells were placed into upper transwell chambers (BD Biosciences) with 0.3 mL serum-free medium and 0.5 mL medium with 10% FBS was added to the lower chambers of the 24-well plate (Corning). After incubating for 48 h at 37 °C in 5% CO_2_, the migrating cells adhered to the bottom of the chambers were fixed with 20% methanol for 20 min. Staining cells with 0.1% crystal violet (Sangon Biotech) and then removing cells in the upper surface of inserts with cotton swabs. The stained cells were photographed and counted under a microscope in 8 fields with random choice.

### Colony formation assay

For colony formation assay, 5000 cells were plated into the six-well plate for 2 weeks. Colonies were fixed with 4% formaldehyde for 20 min and stained with 0.1% crystal violet for 1 h. The number of colonies with more than 50 cells was counted and normalized to the untreated group.

### Patient samples

Fresh-frozen primary HCC tissues and their paired normal samples were obtained from patients undergoing surgical resection at Zhuhai People’s Hospital (Zhuhai, China) after consent was obtained from the patients. None of the patients received any prior radiochemotherapy. For total protein extraction, place an equal amount of tissues (40 mg) in tubes and grind the tissue with a plastic rod for 50–60 times with twisting force on the ice. Then, add five times cell lysis buffer (50 mM Tris pH 8.0, 0.1 M NaCl, 10 mM NaF, 1 mM Na_3_VO_4_, 0.5% NP-40, 10% Glycerol and 1 mM EDTA pH 8.0) and continue to grind for 50–60 times. Cap the tube and incubate on ice for 10–15 min. Centrifuge at 12,000 rpm for 15 min. The supernatant was subject to IB assay following standard protocols. This study was approved by the Ethics Committee of Zhuhai People’s Hospital (approval number: 2022-53). Before tissue acquisition, written informed consent was obtained from each patient.

### Statistical analysis

The density of the immunoblotting band was quantified by Image J software. All statistical analyses were performed with GraphPad Prism software (GraphPad Software Inc., La Jolla, CA). The data shown in this study were representative of at least three times independent replicates and were analyzed by unpaired Student’s *t*-test. *P* < 0.05 was considered statistically significant (*P < 0.05, **P < 0.01, ***P < 0.001 and ****P < 0.0001).

### Supplementary information

reproducibility checklist

Supplementary Information

## Data Availability

All relevant data are available from the corresponding author upon reasonable request.
