# Supplementary material for: SPOP point mutations regulate substrate preference and affect its function
Source: Cell Death Dis. 2024 Feb 26;15(2):172. doi: 10.1038/s41419-024-06565-1 (PMC10897488; doi:10.1038/s41419-024-06565-1)
Supplement: Supplementary file 2 — Supplementary Information [file 41419_2024_6565_MOESM2_ESM.pdf]

Supplementary Information

SPOP point mutations regulate substrate preference and affect its function

Yanran Deng et al.

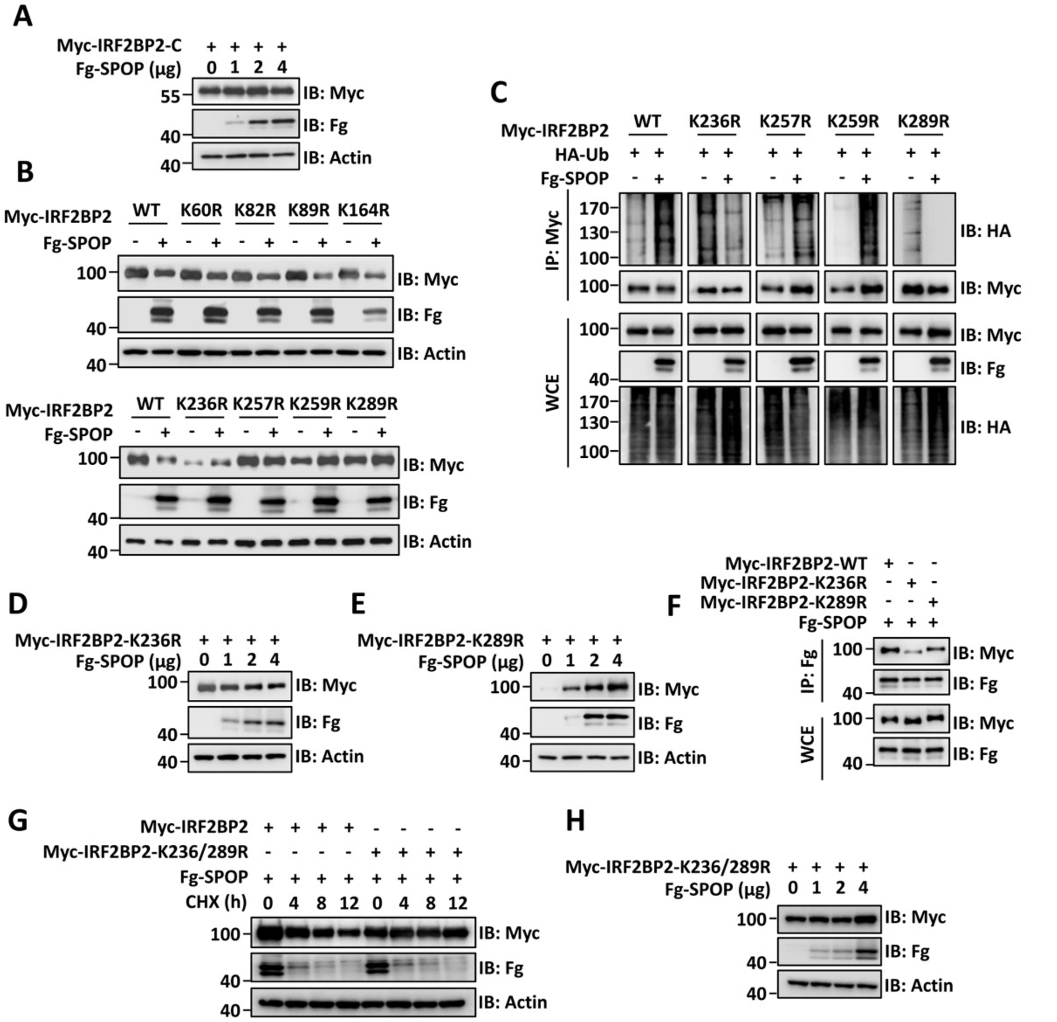

Figure S1. SPOP-mediated IRF2BP2 ubiquitination mainly occurs at K236 and K289

(**A**) Fg-SPOP failed to degrade Myc-IRF2BP2-C protein in HEK-293T cells. (**B**) Immunoblotting of indicated proteins from HEK-293T cells transfected with the indicated plasmids. (**C**) Fg-SPOP did not promote the ubiquitination of Myc-IRF2BP2-K236R and Myc-IRF2BP2-K289R. (**D**) Fg-SPOP failed to degrade Myc-IRF2BP2-K236R protein. (**E**) Fg-SPOP did not decrease Myc-IRF2BP2-K289R protein. (**F**) The

co-IP assays showed the interaction between SPOP and IRF2BP2, IRF2BP2-K236R or IRF2BP2-K289R. (**G**) Protein levels of Myc-IRF2BP2 or Myc-IRF2BP2-K236/289R from HEK-293T cells co-transfected with Fg-SPOP and treated with CHX for indicated intervals. (**H**) Fg-SPOP failed to degrade Myc-IRF2BP2-K236/289R protein. For all results, Actin acts as a loading control.

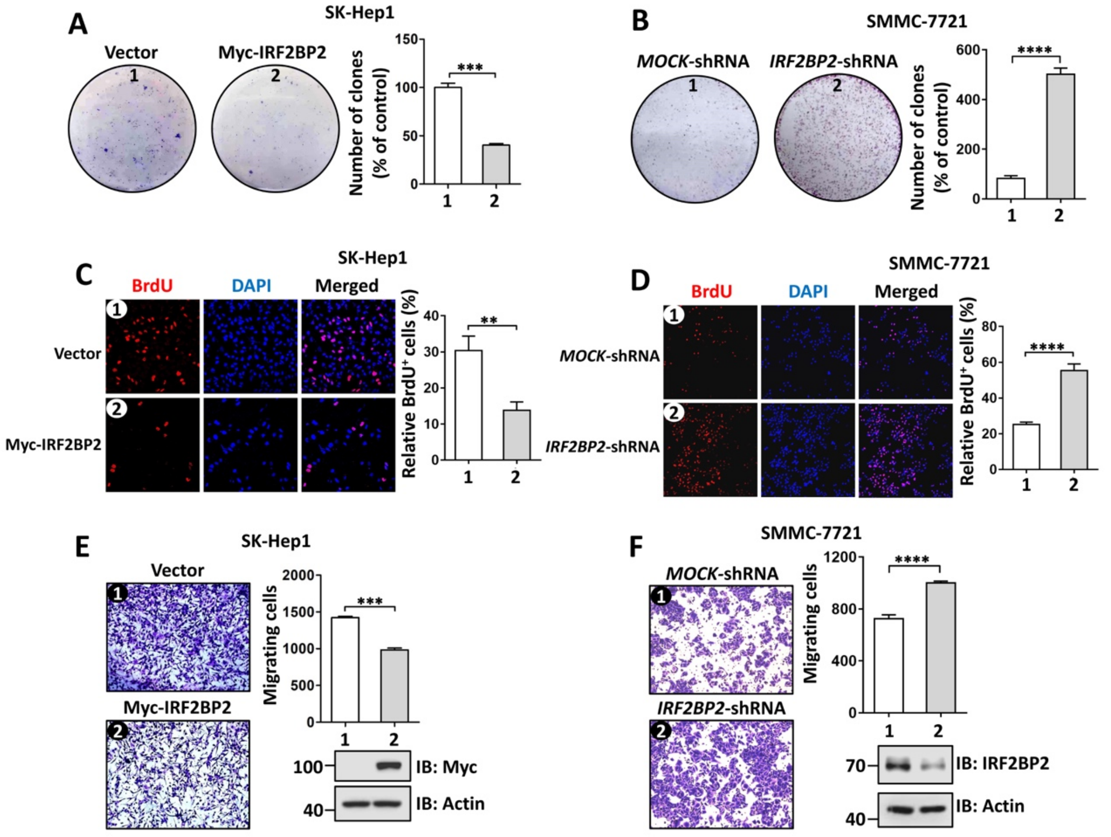

Figure S2. IRF2BP2 plays tumor suppressive roles in HCC cells

(**A**) The colony formation assays showed that overexpression of IRF2BP2 inhibited SK-Hep1 cell proliferation. Quantitative analyses were shown on the right. (**B**) Knockdown of IRF2BP2 promoted SMMC-7721 cell proliferation. Quantitative analyses were shown on the right. (**C**) The BrdU incorporation assays revealed that overexpression of IRF2BP2 suppressed SK-Hep1 cell proliferation. Quantitative analyses were shown on the right. (**D**) Knockdown of IRF2BP2 elevates BrdU incorporation in SMMC-7721 cells. Quantitative analyses were shown on the right. (**E**) Overexpression of IRF2BP2 inhibited SK-Hep1 cell migration. Protein expressions and quantitative analyses were shown on the right. (**F**) Knockdown of IRF2BP2 promoted SMMC-7721 cell migration.

Protein expressions and quantitative analyses were shown on the right. For statistical results, data are shown as means±SEM from three biological-independent repeats. In all above, $**P < 0.01$, $***P < 0.001$, $****P < 0.0001$ based on student's t-test.

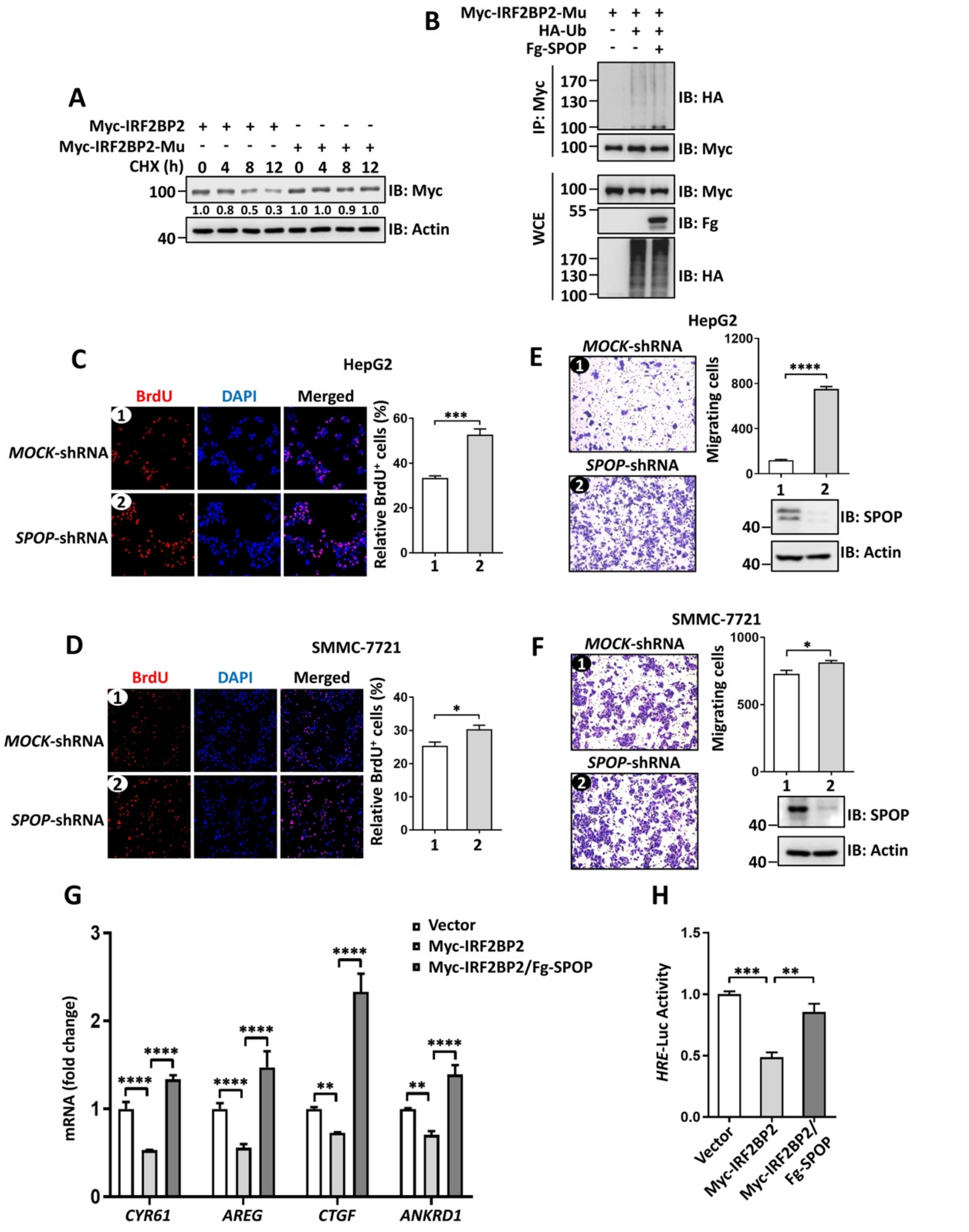

Figure S3. Knockdown of SPOP promotes HCC cell proliferation and migration

(**A**) Protein levels of Myc-IRF2BP2 or Myc-IRF2BP2-Mu from HEK-293T cells treated with CHX for indicated intervals. Actin acts as a loading control. (**B**) SPOP

failed to promote IRF2BP2-Mu ubiquitination. (**C**-**D**) Knockdown of SPOP enhanced BrdU incorporation of HepG2 cells (C) and SMMC-7721 cells (D). Quantitative analyses were shown on the right. (**E**-**F**) Knockdown of SPOP promoted migration of HepG2 cells (E) and SMMC-7721 cells (F). Protein expressions and quantitative analyses were shown on the right. (**G**) The relative mRNA levels of YAP target genes were detected by RT-PCR. (**H**) SPOP rescued IRF2BP2-induced *HRE*-Luc reduction. In all above, *$P < 0.05$, ***$P < 0.001$ and ****$P < 0.0001$ based on student's t-test.

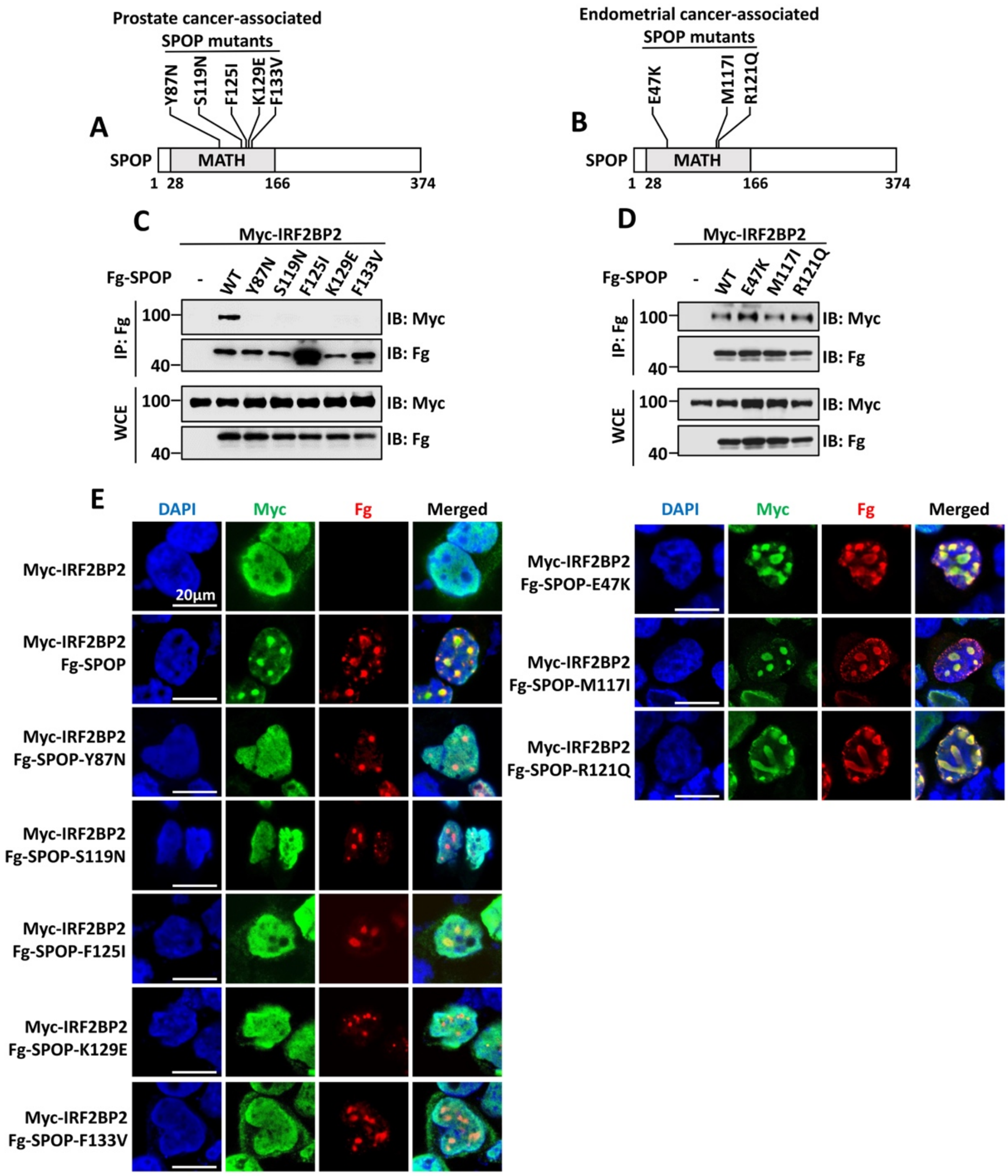

Figure S4. Tumor-associated SPOP mutations affect its affinity to IRF2BP2

(**A**) Point mutations in SPOP protein from prostate cancers. (**B**) Point mutations in

SPOP protein from endometrial cancers. (**C**) Prostate cancer-derived SPOP mutants could not pull down Myc-IRF2BP2. (**D**) Endometrial cancer-associated SPOP mutants were able to bind Myc-IRF2BP2. (**E**) HEK-293T cells expressing indicated constructs were stained to show the co-localization of IRF2BP2 and SPOP mutants. DAPI staining marks cell nuclei. Scale bars: 20 μm for all images.

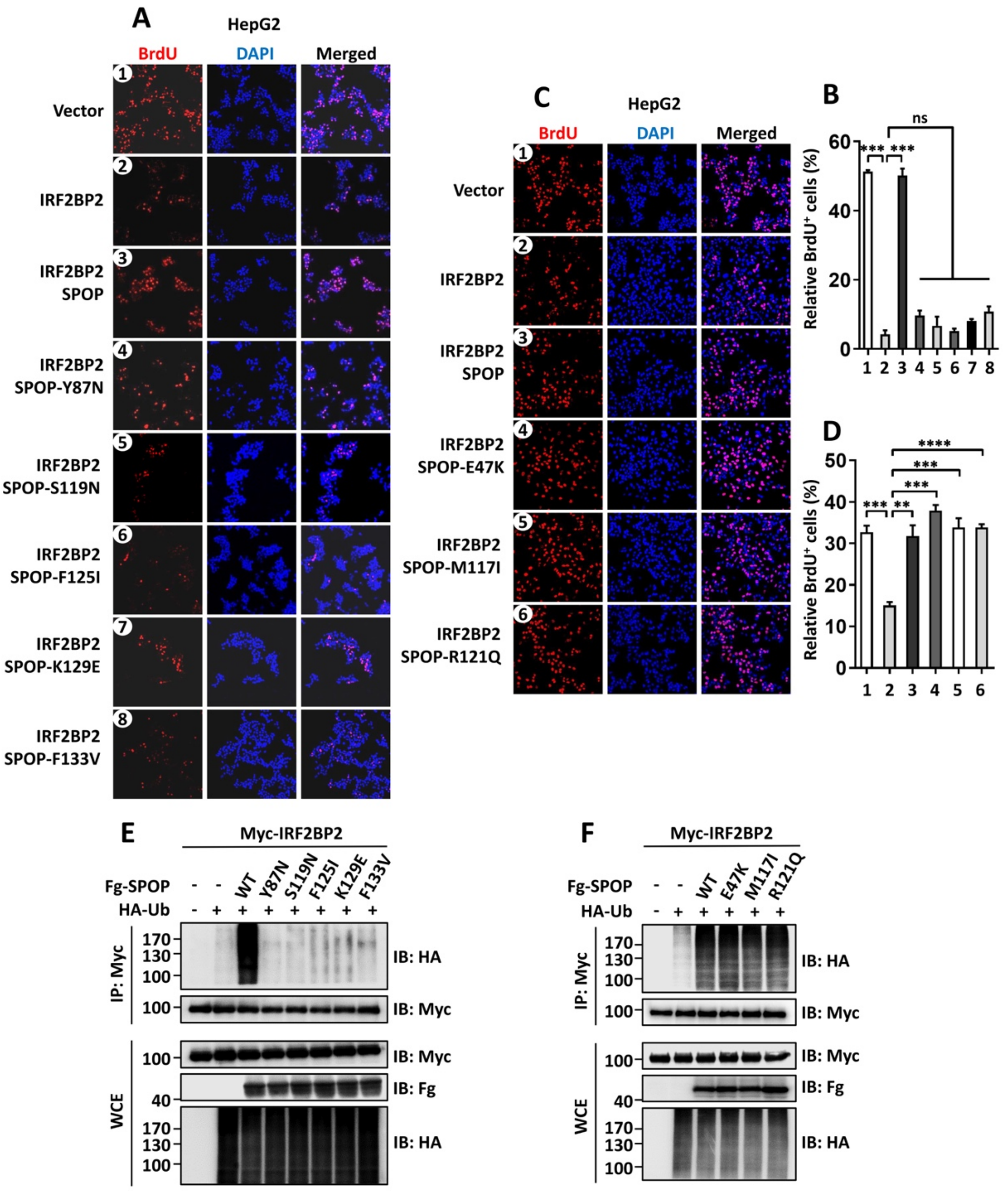

Figure S5. Effects of PC- and EC-associated SPOP mutants on IRF2BP2-induced inhibition of cell proliferation

(**A**-**B**) The BrdU incorporation assays revealed that PC-associated SPOP mutants were unable to restore the anti-proliferation role of IRF2BP2 in HepG2 cells. (**C**-**D**) EC-

derived SPOP mutants could reverse the anti-proliferation role of IRF2BP2 in HepG2 cells. (**E**) Prostate cancer-derived SPOP mutants could not promote ubiquitination of Myc-IRF2BP2. (**F**) Endometrial cancer-associated SPOP mutants were able to promote ubiquitination of Myc-IRF2BP2. In all above, $**P < 0.01$, $***P < 0.001$, $****P < 0.0001$, ns: not significant difference based on student's *t*-test.

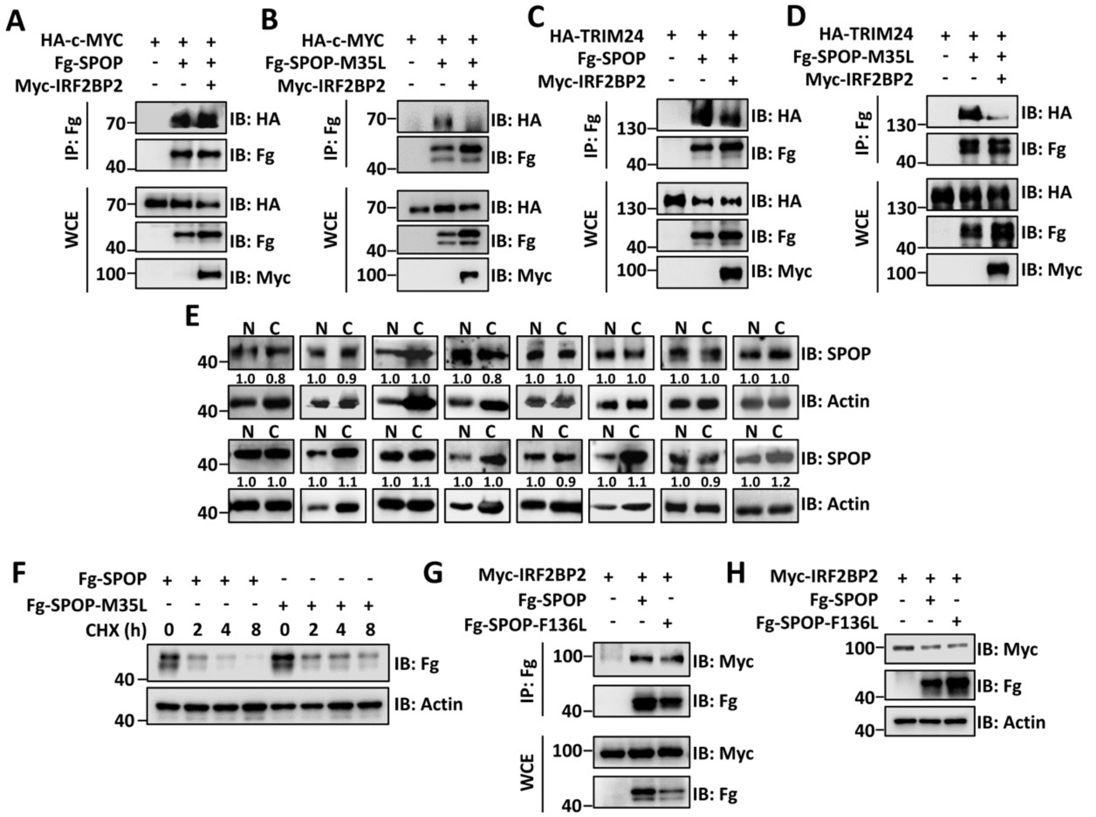

Figure S6. SPOP-M35L prefers binding IRF2BP2

(**A**) IRF2BP2 did not affect SPOP binding to c-MYC. (**B**) IRF2BP2 weakened the interaction between SPOP-M35L and c-MYC. (**C**) IRF2BP2 failed to influence SPOP-TRIM24 interaction. (**D**) IRF2BP2 enabled to decrease the affinity of TRIM24 to SPOP-M35L. (**E**) The protein levels of SPOP from liver cancer samples and corresponding para-cancer normal samples. Of note, SPOP proteins have no changes in cancers. Relative intensities of SPOP bands were measured using Image J, and normalized to Actin. (**F**) Protein levels of Fg-SPOP or Fg-SPOP-M35L from HEK-293T cells treated with CHX for indicated intervals. (**G**) Fg-SPOP and Fg-SPOP-F136L showed equal affinities to Myc-IRF2BP2 (**H**) Fg-SPOP and Fg-SPOP-F136L showed comparable ability to degrade Myc-IRF2BP2. Above all, Actin acts as a loading control.

Original WB figures:

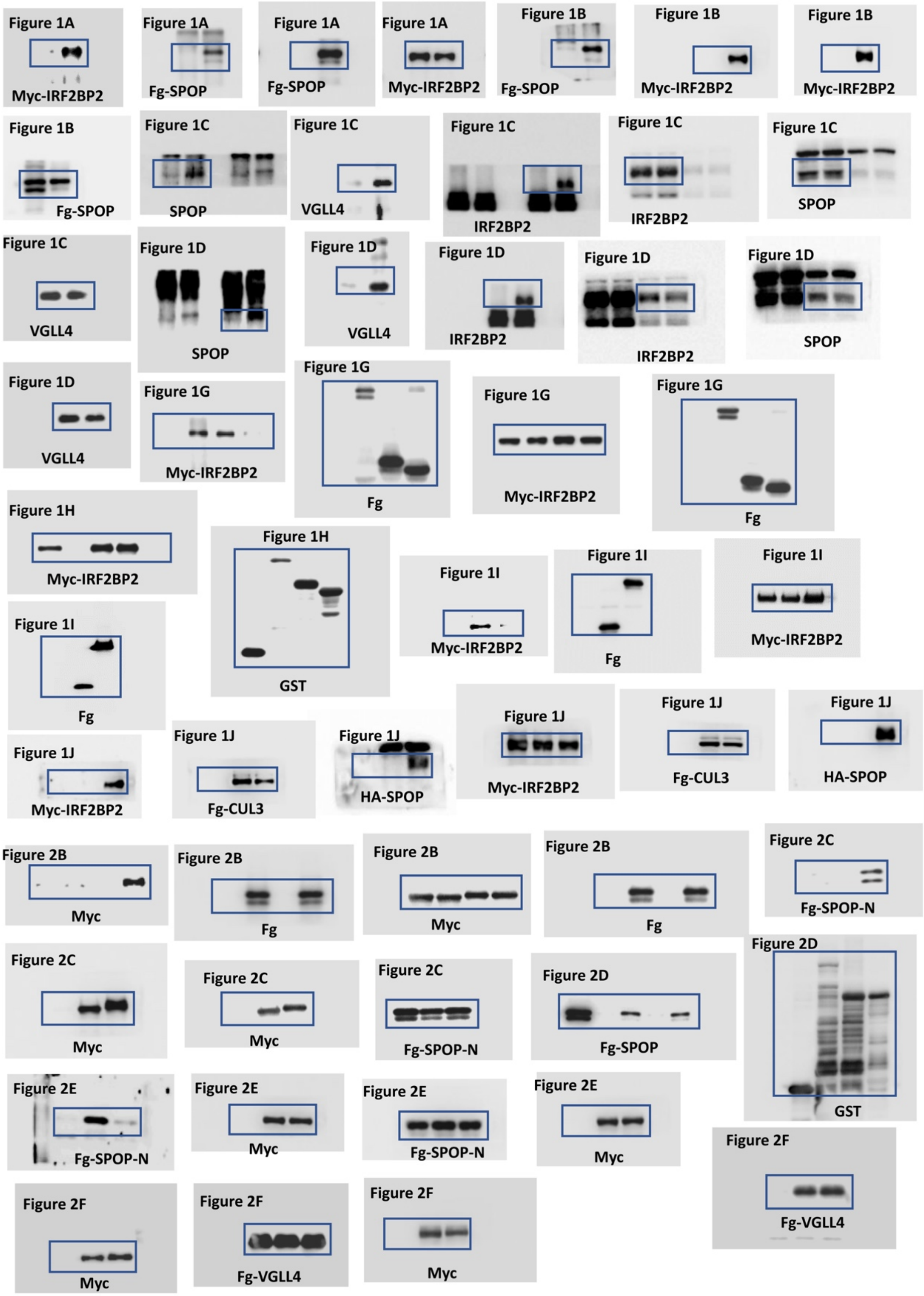

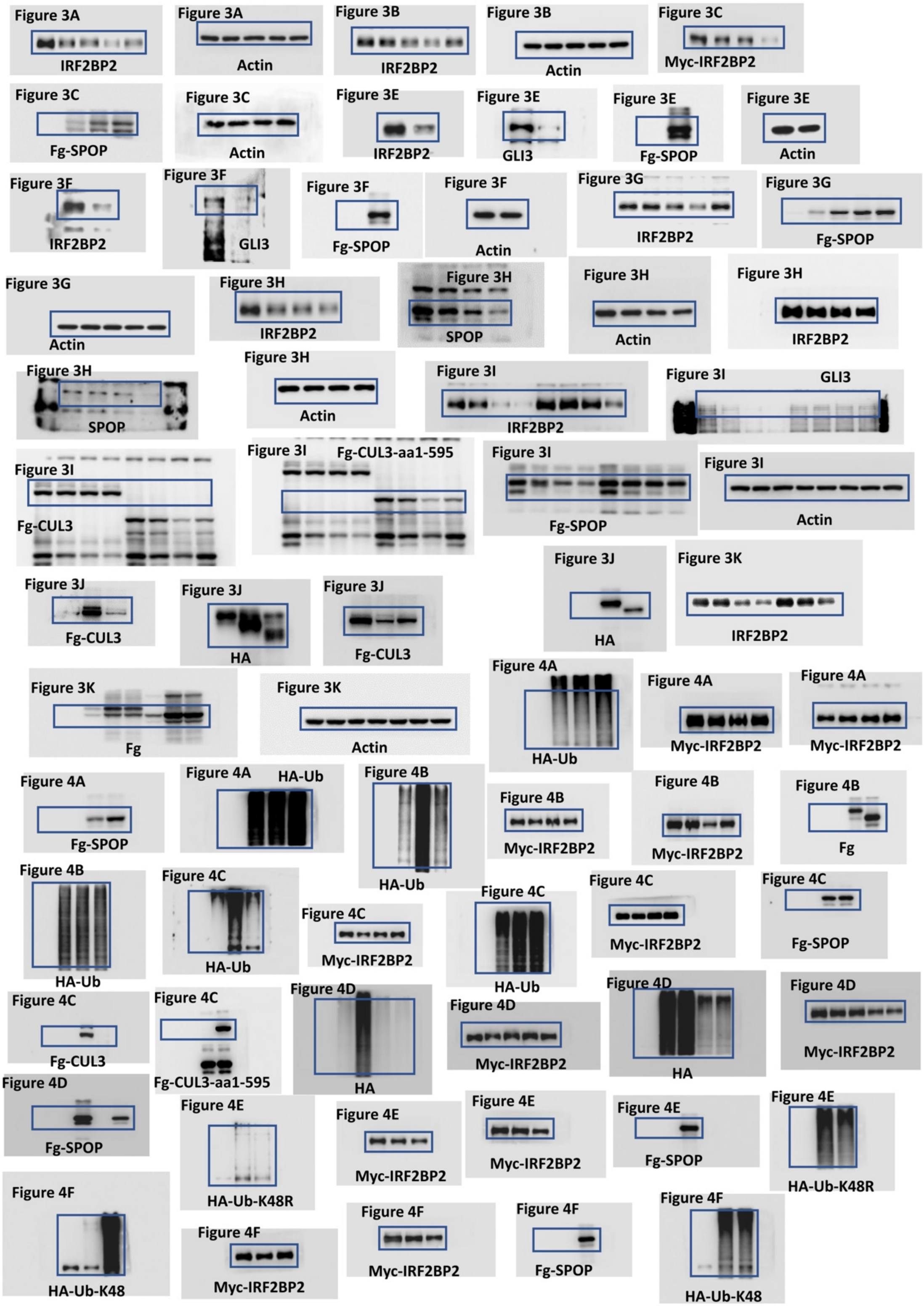
Figure 3A
IRF2BP2
Figure 3A
Actin
Figure 3B
IRF2BP2
Figure 3B
Actin
Figure 3C
Myc-IRF2BP2
Figure 3C
Fg-SPOP
Figure 3C
Actin
Figure 3E
IRF2BP2
Figure 3E
GLI3
Figure 3E
Fg-SPOP
Figure 3E
Actin
Figure 3F
IRF2BP2
Figure 3F
GLI3
Figure 3F
Fg-SPOP
Figure 3F
Actin
Figure 3G
IRF2BP2
Figure 3G
Fg-SPOP
Figure 3G
Actin
Figure 3H
IRF2BP2
Figure 3H
SPOP
Figure 3H
Actin
Figure 3H
IRF2BP2
Figure 3H
SPOP
Figure 3H
Actin
Figure 3I
IRF2BP2
Figure 3I
GLI3
Figure 3I
Fg-CUL3
Figure 3I
Fg-CUL3-aa1-595
Figure 3I
Fg-SPOP
Figure 3I
Actin
Figure 3J
HA
Figure 3K
IRF2BP2
Figure 3J
Fg-CUL3
Figure 3J
HA
Figure 3J
Fg-CUL3
Figure 4A
HA-Ub
Figure 4A
Myc-IRF2BP2
Figure 4A
Myc-IRF2BP2
Figure 3K
Fg
Figure 3K
Actin
Figure 4A
Fg-SPOP
Figure 4A
HA-Ub
Figure 4B
HA-Ub
Figure 4B
Myc-IRF2BP2
Figure 4B
Myc-IRF2BP2
Figure 4B
Fg
Figure 4B
HA-Ub
Figure 4C
HA-Ub
Figure 4C
Myc-IRF2BP2
Figure 4C
HA-Ub
Figure 4C
Myc-IRF2BP2
Figure 4C
Fg-SPOP
Figure 4C
Fg-CUL3
Figure 4C
Fg-CUL3-aa1-595
Figure 4D
HA
Figure 4D
Myc-IRF2BP2
Figure 4D
HA
Figure 4D
Myc-IRF2BP2
Figure 4D
Fg-SPOP
Figure 4E
HA-Ub-K48R
Figure 4E
Myc-IRF2BP2
Figure 4E
Myc-IRF2BP2
Figure 4E
Fg-SPOP
Figure 4E
HA-Ub-K48R
Figure 4F
HA-Ub-K48
Figure 4F
Myc-IRF2BP2
Figure 4F
Myc-IRF2BP2
Figure 4F
Fg-SPOP
Figure 4F
HA-Ub-K48

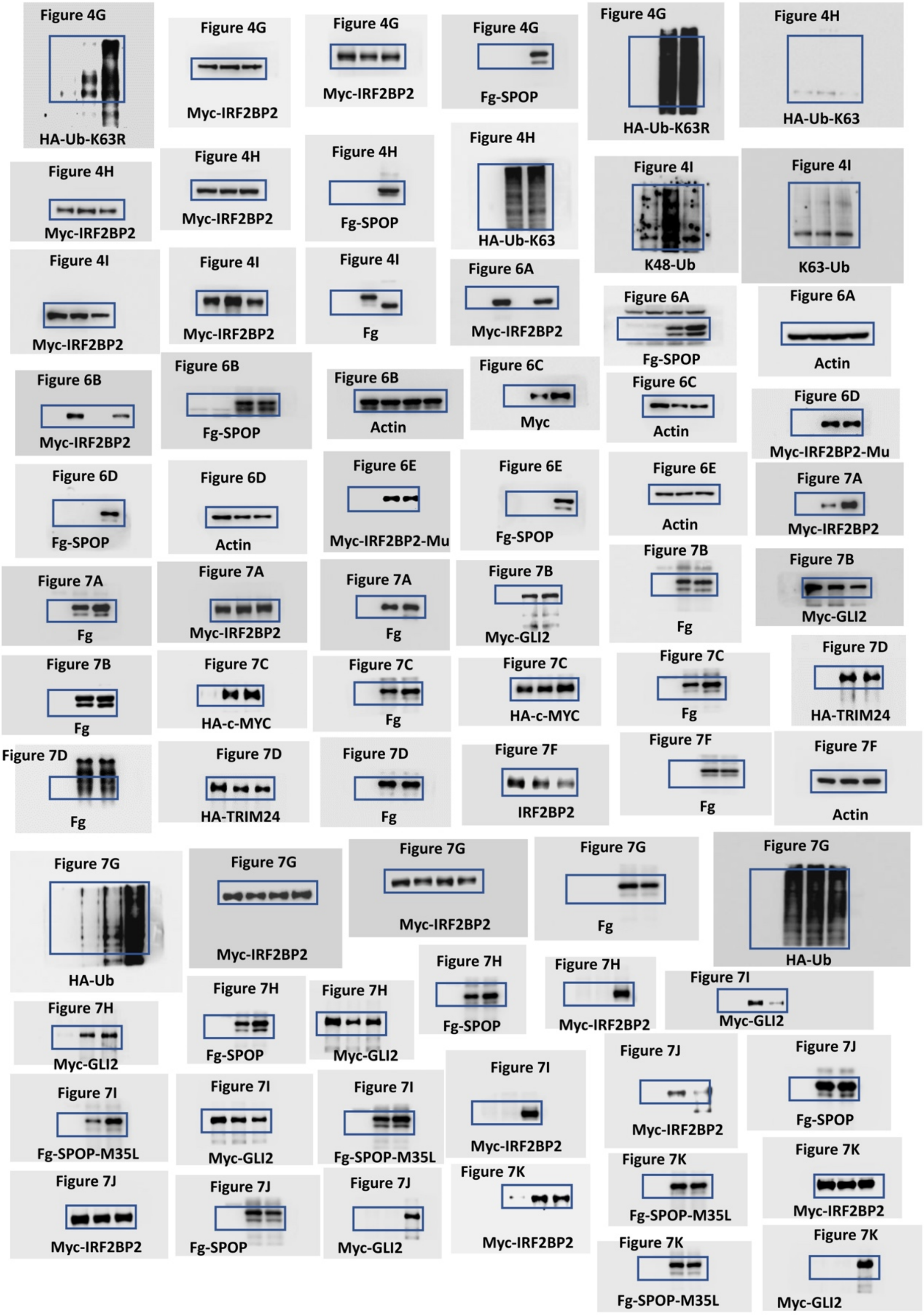
Figure 4G
HA-Ub-K63R
Figure 4G
Myc-IRF2BP2
Figure 4G
Myc-IRF2BP2
Figure 4G
Fg-SPOP
Figure 4G
HA-Ub-K63R
Figure 4H
HA-Ub-K63
Figure 4H
Myc-IRF2BP2
Figure 4H
Myc-IRF2BP2
Figure 4H
Fg-SPOP
Figure 4H
HA-Ub-K63
Figure 4I
K48-Ub
Figure 4I
K63-Ub
Figure 4I
Myc-IRF2BP2
Figure 4I
Myc-IRF2BP2
Figure 4I
Fg
Figure 6A
Myc-IRF2BP2
Figure 6A
Fg-SPOP
Figure 6A
Actin
Figure 6B
Myc-IRF2BP2
Figure 6B
Fg-SPOP
Figure 6B
Actin
Figure 6C
Myc
Figure 6C
Actin
Figure 6D
Myc-IRF2BP2-Mu
Figure 6D
Fg-SPOP
Figure 6D
Actin
Figure 6E
Myc-IRF2BP2-Mu
Figure 6E
Fg-SPOP
Figure 6E
Actin
Figure 7A
Myc-IRF2BP2
Figure 7A
Fg
Figure 7A
Myc-IRF2BP2
Figure 7A
Fg
Figure 7B
Myc-GLI2
Figure 7B
Fg
Figure 7B
Myc-GLI2
Figure 7B
Fg
Figure 7C
HA-c-MYC
Figure 7C
Fg
Figure 7C
HA-c-MYC
Figure 7C
Fg
Figure 7D
HA-TRIM24
Figure 7D
Fg
Figure 7D
HA-TRIM24
Figure 7D
Fg
Figure 7F
IRF2BP2
Figure 7F
Fg
Figure 7F
Actin
Figure 7G
HA-Ub
Figure 7G
Myc-IRF2BP2
Figure 7G
Myc-IRF2BP2
Figure 7G
Fg
Figure 7G
HA-Ub
Figure 7H
Myc-GLI2
Figure 7H
Fg-SPOP
Figure 7H
Myc-GLI2
Figure 7H
Fg-SPOP
Figure 7H
Myc-IRF2BP2
Figure 7I
Myc-GLI2
Figure 7I
Fg-SPOP-M35L
Figure 7I
Myc-GLI2
Figure 7I
Fg-SPOP-M35L
Figure 7I
Myc-IRF2BP2
Figure 7J
Myc-IRF2BP2
Figure 7J
Fg-SPOP
Figure 7J
Myc-IRF2BP2
Figure 7J
Fg-SPOP
Figure 7J
Myc-GLI2
Figure 7K
Myc-IRF2BP2
Figure 7K
Fg-SPOP-M35L
Figure 7K
Myc-IRF2BP2
Figure 7K
Fg-SPOP-M35L
Figure 7K
Myc-GLI2

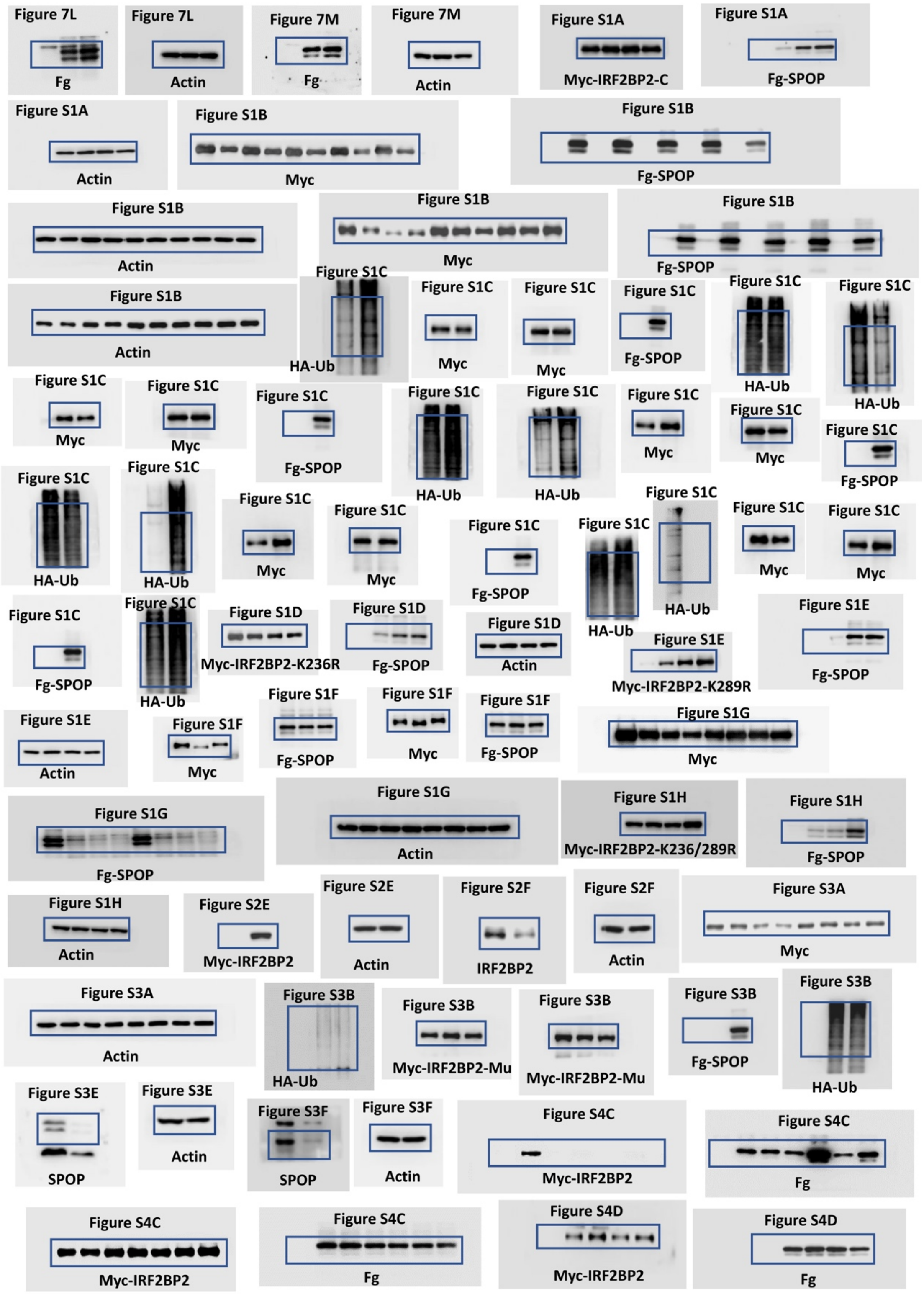
Figure 7L
Fg
Figure 7L
Actin
Figure 7M
Fg
Figure 7M
Actin
Figure S1A
Myc-IRF2BP2-C
Figure S1A
Fg-SPOP
Figure S1A
Actin
Figure S1B
Myc
Figure S1B
Fg-SPOP
Figure S1B
Actin
Figure S1B
Myc
Figure S1B
Fg-SPOP
Figure S1B
Actin
Figure S1C
HA-Ub
Figure S1C
Myc
Figure S1C
Myc
Figure S1C
Fg-SPOP
Figure S1C
HA-Ub
Figure S1C
HA-Ub
Figure S1C
Myc
Figure S1C
Myc
Figure S1C
Fg-SPOP
Figure S1C
HA-Ub
Figure S1C
HA-Ub
Figure S1C
Myc
Figure S1C
Myc
Figure S1C
Fg-SPOP
Figure S1C
HA-Ub
Figure S1C
HA-Ub
Figure S1C
Myc
Figure S1C
Myc
Figure S1C
Fg-SPOP
Figure S1C
HA-Ub
Figure S1C
HA-Ub
Figure S1C
Myc
Figure S1C
Myc
Figure S1C
Fg-SPOP
Figure S1C
HA-Ub
Figure S1D
Myc-IRF2BP2-K236R
Figure S1D
Fg-SPOP
Figure S1D
Actin
Figure S1E
Myc-IRF2BP2-K289R
Figure S1E
Fg-SPOP
Figure S1E
Actin
Figure S1F
Myc
Figure S1F
Fg-SPOP
Figure S1F
Myc
Figure S1F
Fg-SPOP
Figure S1G
Myc
Figure S1G
Fg-SPOP
Figure S1G
Actin
Figure S1H
Myc-IRF2BP2-K236/289R
Figure S1H
Fg-SPOP
Figure S1H
Actin
Figure S2E
Myc-IRF2BP2
Figure S2E
Actin
Figure S2F
IRF2BP2
Figure S2F
Actin
Figure S3A
Myc
Figure S3A
Actin
Figure S3B
HA-Ub
Figure S3B
Myc-IRF2BP2-Mu
Figure S3B
Myc-IRF2BP2-Mu
Figure S3B
Fg-SPOP
Figure S3B
HA-Ub
Figure S3E
SPOP
Figure S3E
Actin
Figure S3F
SPOP
Figure S3F
Actin
Figure S4C
Myc-IRF2BP2
Figure S4C
Fg
Figure S4C
Myc-IRF2BP2
Figure S4C
Fg
Figure S4D
Myc-IRF2BP2
Figure S4D
Fg

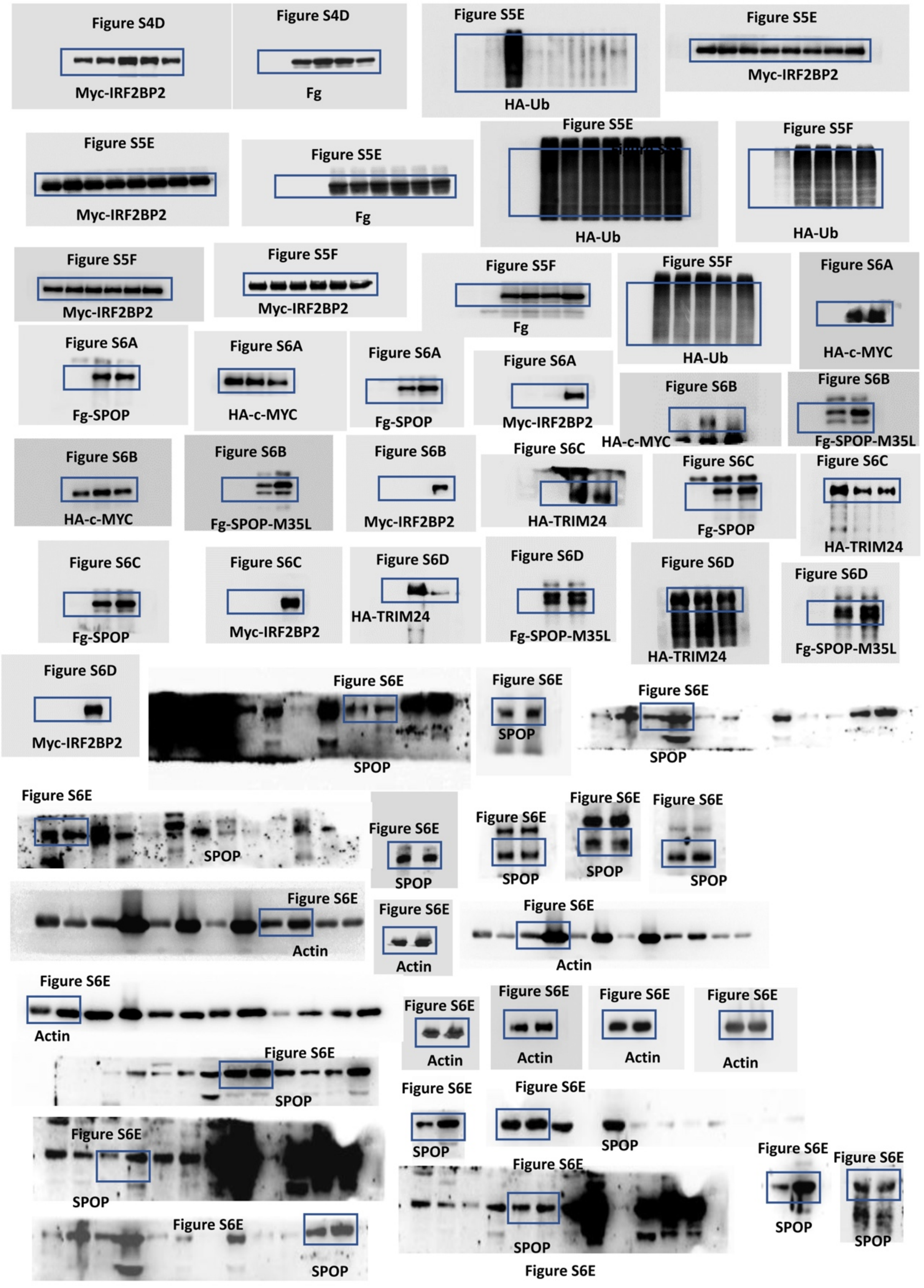
Figure S4D
Myc-IRF2BP2
Figure S4D
Fg
Figure S5E
HA-Ub
Figure S5E
Myc-IRF2BP2
Figure S5E
Myc-IRF2BP2
Figure S5E
Fg
Figure S5E
HA-Ub
Figure S5F
HA-Ub
Figure S5F
Myc-IRF2BP2
Figure S5F
Myc-IRF2BP2
Figure S5F
Fg
Figure S5F
HA-Ub
Figure S6A
HA-c-MYC
Figure S6A
Fg-SPOP
Figure S6A
HA-c-MYC
Figure S6A
Fg-SPOP
Figure S6A
Myc-IRF2BP2
Figure S6B
HA-c-MYC
Figure S6B
Fg-SPOP-M35L
Figure S6B
HA-c-MYC
Figure S6B
Fg-SPOP-M35L
Figure S6B
Myc-IRF2BP2
Figure S6C
HA-TRIM24
Figure S6C
Fg-SPOP
Figure S6C
HA-TRIM24
Figure S6C
Fg-SPOP
Figure S6C
Myc-IRF2BP2
Figure S6D
HA-TRIM24
Figure S6D
Fg-SPOP-M35L
Figure S6D
HA-TRIM24
Figure S6D
Fg-SPOP-M35L
Figure S6D
Myc-IRF2BP2
Figure S6E
SPOP
Figure S6E
SPOP
Figure S6E
SPOP
Figure S6E
SPOP
Figure S6E
SPOP
Figure S6E
SPOP
Figure S6E
SPOP
Figure S6E
SPOP
Figure S6E
Actin
Figure S6E
Actin
Figure S6E
Actin
Figure S6E
Actin
Figure S6E
Actin
Figure S6E
Actin
Figure S6E
Actin
Figure S6E
Actin
Figure S6E
SPOP
Figure S6E
SPOP
Figure S6E
SPOP
Figure S6E
SPOP
Figure S6E
SPOP
Figure S6E
SPOP
Figure S6E
SPOP
Figure S6E
SPOP
Figure S6E

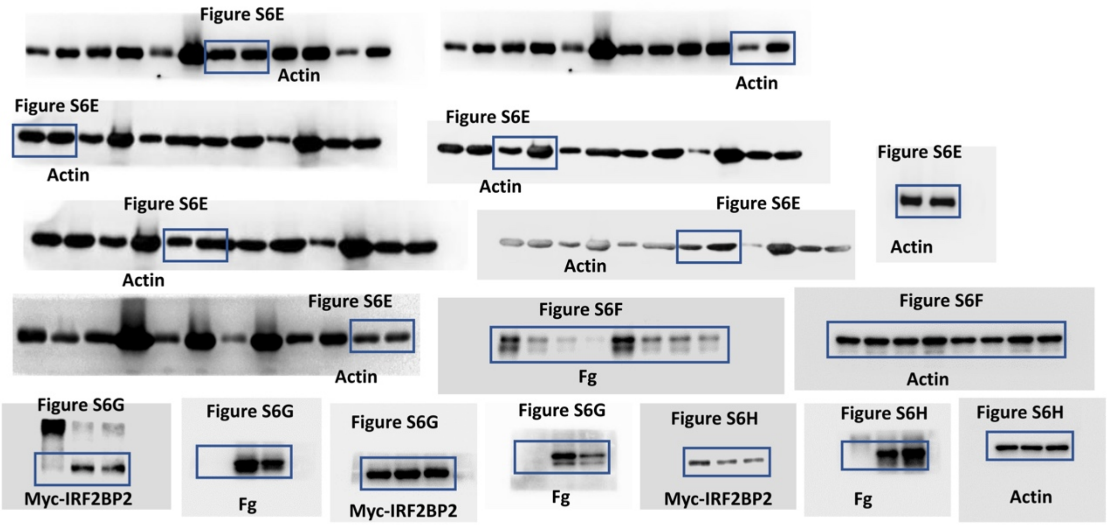
Figure S6E
Actin
Actin
Figure S6E
Actin
Figure S6E
Actin
Figure S6E
Actin
Figure S6E
Actin
Figure S6E
Actin
Figure S6E
Actin
Figure S6F
Fg
Figure S6F
Actin
Figure S6G
Myc-IRF2BP2
Figure S6G
Fg
Figure S6G
Myc-IRF2BP2
Figure S6G
Fg
Figure S6H
Myc-IRF2BP2
Figure S6H
Fg
Figure S6H
Actin
